# Emerging Memtransistors for Neuromorphic System Applications: A Review

**DOI:** 10.3390/s23125413

**Published:** 2023-06-07

**Authors:** Tao You, Miao Zhao, Zhikang Fan, Chenwei Ju

**Affiliations:** 1 High-Frequency High-Voltage Device and Integrated Circuits R&D Center, Institute of Microelectronics of the Chinese Academy of Sciences, 3 Beitucheng West Road, Beijing 100029, China; youtao21@mails.ucas.ac.cn (T.Y.);; 2 University of Chinese Academy of Sciences, Beijing 100029, China

**Keywords:** memtransistors, neuromorphic behavior, ferroelectric material, resistive switching

## Abstract

The von Neumann architecture with separate memory and processing presents a serious challenge in terms of device integration, power consumption, and real-time information processing. Inspired by the human brain that has highly parallel computing and adaptive learning capabilities, memtransistors are proposed to be developed in order to meet the requirement of artificial intelligence, which can continuously sense the objects, store and process the complex signal, and demonstrate an “all-in-one” low power array. The channel materials of memtransistors include a range of materials, such as two-dimensional (2D) materials, graphene, black phosphorus (BP), carbon nanotubes (CNT), and indium gallium zinc oxide (IGZO). Ferroelectric materials such as P(VDF-TrFE), chalcogenide (PZT), Hf_x_Zr_1−x_O_2_(HZO), In_2_Se_3_, and the electrolyte ion are used as the gate dielectric to mediate artificial synapses. In this review, emergent technology using memtransistors with different materials, diverse device fabrications to improve the integrated storage, and the calculation performance are demonstrated. The different neuromorphic behaviors and the corresponding mechanisms in various materials including organic materials and semiconductor materials are analyzed. Finally, the current challenges and future perspectives for the development of memtransistors in neuromorphic system applications are presented.

## 1. Introduction

With the rapid development of information technologies such as the Internet of Things and artificial intelligence (AI) technologies, it is challenging for conventional von Neumann computing architecture hardware to satisfy the requirements of modern applications. Emerging hardware-based neuromorphic computing structures with a high operating speed, low-power operation, and minimal size-volume devices must be explored in terms of the various materials capable of simulating the brain’s functions. These structures are inspired by the human brain’s highly parallel computing and adaptive learning capabilities [1,2,3]. In 1971, Cai hypothesized that, in addition to resistance, capacitance, and inductance, there should be a fourth fundamental element in nature: the memristor, which depicts the interaction between the magnetic flux and the charge via the element [4]. The element resistance varies based on the charge that has previously flowed through it. In 2008, Hewlett-Packard was the first company to develop nano memristors; the scientific community has since witnessed an increasing wave of memristor research and development [5]. The memristor is one of the most efficient techniques to implement in nonvolatile memory, which is advantageous for enhancing circuit integration. Memristors are regarded as the best approach to implementing large-scale artificial neural networks due to their unique nonlinear characteristics [6,7,8,9]. Traditional two-terminal memristor devices, however, lack extra bias ports for controlling the conductivity response of the devices, so in the cross array, a selection device (such as a selection transistor and memristor switch) is required to program a single node, which makes the circuit integration process more complex and limits the integration density. In this instance, the emergence of a memtransistor with an adjustable gate presents scientists with a novel concept. Memtransistors are three-terminal devices with channel material stacks, the channel conductance is modulated by the gate, in which the gate metals are regarded as the bottom electrode (BE) and the top electrode (TE), and the channel resistance changes with the gate voltages [10,11]. In a single memtransistor, it combines the memory resistance properties of the memristor with the switching characteristics. By controlling the third end, it is possible to achieve continuous control over the increase and decrease in the device’s conductance. Through the selection of the component’s channel material, the component’s leakage current can be reduced to a lower level. This not only improves the accuracy of integrated circuits, but also satisfies the requirements of circuit integration, and has a promising future application in terms of low power consumption and high AI computing level [12,13,14]. Emerging memtransistors are used to realize an effective emulation of a neuromorphic system in hardware terms. The device has various advantages, such as non-volatility, a simple structure, low power, and miniaturization. In recent years, neuromorphic computing technologies with resistive switching mechanisms have been investigated, and the different materials and various device fabrications have been explored [15,16,17,18].

A variety of emerging devices have been proposed in order to realize neuromorphic behaviors. Resistive random-access memory (RRAM) [19,20,21], phase-change random access memory (PCRAM) [22], and magnetic random-access memory (MRAM) [23] have been adapted by researchers. In recent years, emerging memtransistors have been considered a promising technology, an alternative technical route in the future of neuromorphic computing systems. The memtransistor combines memory and processing into one single device that is similar to the biological synapse. As we know, the synapse has an important role in the behavior of learning and memory; according to estimates, the human brain contains 10^11^ neurons and 10^15^ synapses, of which synapses are crucial components of the nervous system and connect neurons; simulating synapses with a single component is an efficient way to create a new computing architecture that functions like the brain [24,25,26,27]. Presynaptic membranes, postsynaptic membranes, and synaptic gaps make up biological synapses. By stimulating them with appropriate action potential, it is possible to regulate the release of relevant neurotransmitters, leading to phenomena such as postsynaptic excitation and postsynaptic inhibition [28,29,30,31,32]. Additionally, the precise modulation of the synapse weight (the strength of the synaptic connections) by presynaptic/postsynaptic action potentials is referred to as synaptic plasticity. Therefore, the memtransistor should demonstrate the weight modulation (conductance) performance, and it is essential that the device exhibits biological behavior, which confirms the memtransistor can be used in the neuromorphic computing application [32,33,34,35,36,37,38].

In this review, different channel materials such as 2D materials, graphene, BP, carbon nanotubes (CNT) and indium gallium zinc oxide (IGZO) are discussed. At the same time, ferroelectric materials such as poly(vinylidenefluoride-co-trifluoroethylene) (P(VDF-TrFE)), chalcogenide (Pb (Zr,Ti) O_3_), Hf_x_Zr_1-x_O_2_(HZO), In_2_Se_3_, and the electrolyte ion-gate are also demonstrated. Various emerging engineering device fabrications are investigated to improve the device capabilities in in-memory-computing hardware for neuromorphic system applications [39,40,41,42]. We focus on different materials, different device fabrications, and different electrical performances regarding the memtransistors. In Section 2, the resistive switching (RS) mechanisms and properties are discussed. In Section 3, the 2D-materials-based memtransistor constructed for the neuromorphic system is presented. In Section 4, the ferroelectric field-effect transistors are demonstrated, including the inorganic ferroelectric gate, organic ferroelectric materials, and the 2D ferroelectric materials gate. In Section 5, we focus on the electrolyte ion-gated field-effect transistors. In Section 6 and Section 7, the future applications of memtransistors and the current challenges they present are discussed.

## 2. The Resistive Switching (RS) Mechanisms and Properties of Memtransistors

This section discusses the memtransistors based on various modulation principles. The features of a memtransistor vary based on its structure and its manufacturing. It is proven that memtransistors share a consensus regarding their essential electrical features. The characteristics of resistive switching and the conductance modulation processes by the gate are described. In Section 2.4, the various electrical properties of memtransistors are exhibited.

### 2.1. Switching Mechanisms by the Charge Trapping for Stacking 2D Materials Heterostructure Device

The emergence of 2D materials has spurred the development of novel devices, and heterogeneous structures created by stacking 2D materials with other semiconductor materials or several 2D materials are often used in neuromorphic devices. However, memtransistors based on 2D materials are typically susceptible to environmental influences, as the majority of their memory functions rely on the trapping of carriers by inherent or created defects at the material interface to modulate the transistor channel. The retention of the trap state is unpredictable; hence, the charge trapping process typically results in relatively short retention intervals in the device, while changes in other external factors may also have a significant effect on the modulation mechanism [43,44].

**Figure 1:**
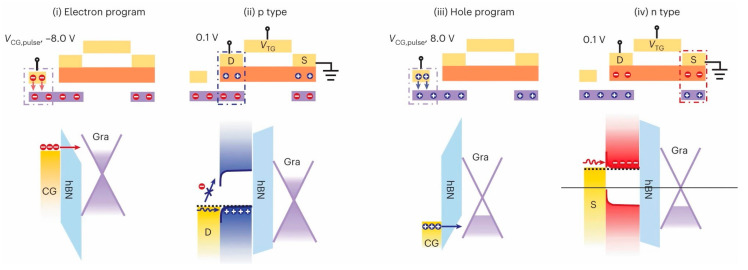
Principle of formation for a bipolar transistor [45]. Copyright © 2022, Xingxia Sun et al., under exclusive license to Springer Nature Limited.

The mechanism of trap charge capture is under the modulation of the applied electric field, which adjusts the energy band of the channel, trap layer, and tunneling layer (dielectric layer), allowing electrons or holes to migrate between different material layers or through the thinning dielectric layer to reach the trap and be captured by the trap. Because the energy band is often modified by the initial electric field without the effect of an external electric field, it is difficult for the trapped carrier to pass the higher potential barrier and return to its original state, resulting in a degree of nonvolatility. The trapped carriers also produce an electric field that modifies the polarity of electrons or holes on the opposite side of the dielectric layer. As depicted in Figure 1, researchers have introduced a floating gate to optimize the contact and achieve the duality conducting behavior of the transistor; by applying a negative voltage of a certain magnitude to the additional set of floating gates, electrons tunnel through the intermediate h-BN layer into the graphene and remain trapped there, causing holes in the WSe_2_ contact region on the graphene [45]. Thus, a smaller hole potential barrier and a larger electron barrier can be simultaneously obtained at the metal/semiconductor interface, resulting in P-type conductivity in the transistor. N-type conductivity in the transistor can be achieved by applying a positive pressure of a certain amplitude via a phase-diverse process.

### 2.2. Modulation of Ferroelectric Polarization in a Ferroelectric Field Effect Transistor

**Figure 2:**
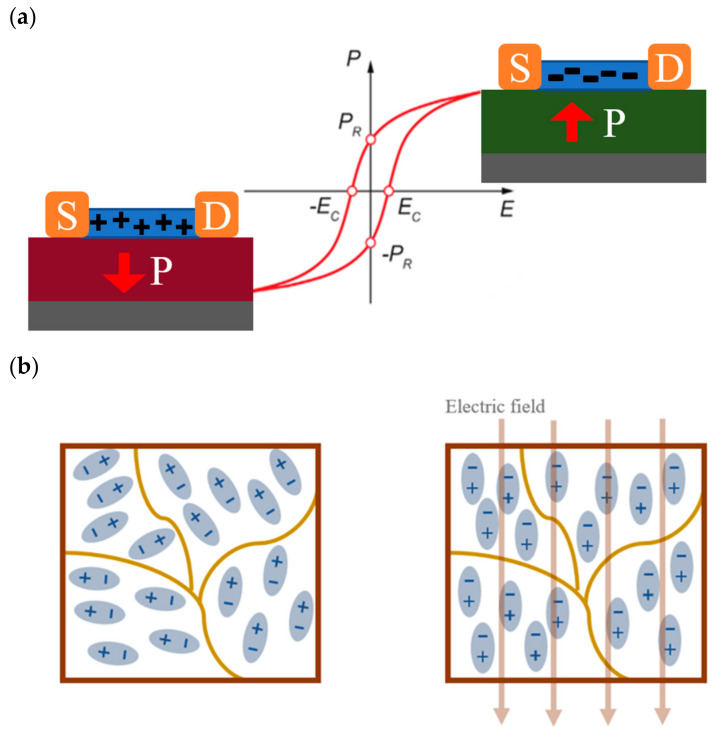
Properties of ferroelectric materials. (**a**) The polarization intensity of ferroelectric materials shows hysteresis curve when the electric field is scanned back and forth [51]. © IOP Publishing. Reproduced with permission. All rights reserved. (**b**) The ferroelectric domains eventually form a stable, uniformly oriented polarization under the external field [52].

In comparison to other regulation systems, ferroelectric polarization regulation controls the degree of ferroelectric dielectric polarization by applying a voltage in order to obtain fine control of channel carriers. Figure 2a depicts a typical ferroelectric hysteresis line, the polarization intensity of which regularly varies with the applied electric field. The two parameter values that merit the most attention from researchers are the remaining polarization value Pr when the electric field is zero, and the electric field value when the polarization is zero (the coercivity field Ec). The former reveals the macroscopic polarization state of the ferroelectric body after the removal of the electric field and can indicate the magnitude of its regulation ability to the outside world, which is an extremely important indicator in the direction of non-volatile memory; the latter indicates the magnitude of the modulation force required for the ferrite polarization flip, which must be carried out with proper consideration in terms of storage and energy efficiency. The working mechanism of the various types of heterostructure transistors mentioned in the previous section is mostly based on the control of trap charge capture/release through the external action of modulating the gate dielectric and channel material barriers to achieve various operating states of the transistor; compared to ferroelectric regulation, this type of regulation mechanism is more uncontrollable and it is difficult for researchers to quantify this type of regulation process in a more subtle way; unlike the various types of ferroelectric-based devices, as shown in Figure 2a, the polarization intensity can be used to measure the regulation strength of ferroelectricity on channel carriers, and the regulation strength has a perfect correspondence with the applied electric field with reference to the hysteresis line, which is more convenient for researchers to regulate the device working state, even though this correspondence can be affected by the interface state, depolarization effect, and internal trap state, but the hysteresis line still gives us a good guide [34,46,47,48,49,50].

**Figure 3:**
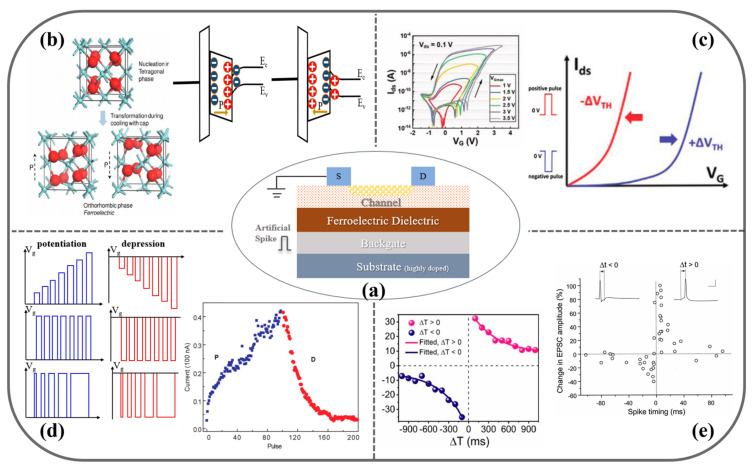
Using ferroelectric transistors to build artificial synapses. (**a**) Schematic of ferroelectric transistor, which has a backgate; (**b**) The polarization state of the ferroelectric layer is adjusted by the external electric field to control the channel carrier density [53,54]. Rights managed by AIP Publishing; (**c**) Threshold voltage shift under ferroelectric polarization regulation and a hysteresis in the transfer curve [55]. © 2020 WILEY−VCH Verlag GmbH & Co. KGaA, Weinheim; (**d**) The scheme of the pulse on the ferroelectric transistor to simulate LTP/LTD [36,56]. Copyright © 2010, American Chemical Society; (**e**) Mechanism of STDP in synapses [57,58]. Copyright © 1998 Society for Neuroscience. Rights managed by AIP Publishing.

When a ferroelectric material is used as the gate dielectric, the orientation of ferroelectric domains in the material tends to be consistent under the regulation of the applied electric field, and finally, a stable ferroelectric polarization field is formed (Figure 2b). In ferroelectric field effect transistors (FeFET), the N-channel material is used as an example, the transistor transfer characteristic curve is tested by applying a regulated electric field at the gate and a reading voltage at the source-drain (Figure 3a). In the process of gradually increasing the gate voltage, the gate dielectric ferroelectric material is regulated by the electric field, and a more stable polarization orientation is formed in the ferroelectric layer during the process (Figure 3b) [53,54], and the shape of the transfer curve is similar to that of the conventional transistor curve; after the gate voltage is applied to the maximum value, the gate control voltage is gradually reduced by means of back sweeping. In the back sweeping process, the transistor transfer curve appears to drift to the left compared with the forward sweeping process. The size of the hysteresis window reflects the gate control capability and the potential for neuromorphic calculation. The key for the formation of the hysteresis window lies in the formation of a stable and effective polarization orientation of the ferroelectric dielectric during the forward sweep (Figure 3c), which generates a corresponding ferroelectric polarization field, and under the regulation of the polarization field, the corresponding carriers are induced in the channel, forming a phenomenon similar to that of depletion transistors, in which the threshold voltage decreases in N-type transistors, while the amount of threshold voltage change is modulated by the magnitude of the gate regulation voltage [55].

By pre-polarization, the ferroelectric dielectric forms a stable polarization field that can regulate the channel, and with the non-volatile nature of the ferroelectric material, the corresponding current value can be observed as a simulation of the PSC process by applying a reading voltage to the source-drain. At the same time, in the long-range modulation process, the repeated application of voltage pulses will enhance the polarization effect in the ferroelectric dielectric, the regulation ability of the channel will be enhanced, and the value of PSC will continue to increase or decrease under the same reading voltage; however, the enhancement of the polarization effect will reach saturation at a certain limit, i.e., the current value will eventually reach saturation, and will not indefinitely increase with the number of pulses. Through these properties, the researchers used ferroelectric transistors to build artificial synapses and modeled some of the basic synaptic biophysics, such as EPSC, PPF, LTP/LTD, and STDP (Figure 3d,e).

### 2.3. Non-Volatile Resistive Switching for Electrolyte Ion-Gated Transistors

The electrolyte ion-gated transistor is a recently proposed three-terminal memristor device with a structure similar to a conventional field effect transistor. Electrolyte ion-gate transistors use electrolytes as the gate dielectric material, where the electrolyte material is characterized by being an insulator of electrons and holes, but a good conductor of ions, such as H^+^, Li^+^, etc. The doping of the channel is achieved by applying different gate voltage regulations to control the electrochemical reaction of the ion migration, thus changing the concentration of carriers in the channel and causing the channel resistance to change, which meets the requirements of a continuous, nonvolatile resistive state change for synaptic devices [59]. The ion dynamics inherent in electrolyte-gated transistors are complex, but in a general analysis of their operation, ions in the electrolyte can be driven towards or even into the channel material at an external voltage, resulting in a change in conductance, and these ion dynamics are very similar to the process of the presynaptic triggering of synaptic vesicles [60,61].

### 2.4. Non-Volatile Resistive Switching for Electrolyte Ion-Gated Transistors

Memtransistors operate as a single synapse that displays memory properties, which emulates the crucial characteristics of the brain in relation to the different materials. The classic electrical characteristics of memtransistors are demonstrated as follows, and some examples are shown in Table 1.

#### 2.4.1. Current Switching Ratio

The current switching ratio of a field effect transistor: the comparison of the current flowing through the channel in the open state of the field effect transistor and the current size flowing through the channel in the closed state, while the source-drain voltage is kept constant.

$$I_{rate}=\frac{I_{on}}{I_{off}}\;$$

#### 2.4.2. Power Consumption Calculation

The energy consumed by the neuromorphic device in a cycle is obtained by integrating the product of current and voltage over the duration of the pulse over time.

$$E=\overset{T_{0}}{\underset{T_{spike}}{\int }}I\times V_{spike}\;∆t$$

#### 2.4.3. Dynamic Range

The ratio of the maximum and minimum conductance values that a synaptic-like neuromorphic device can achieve in long-range modulation.

$$\frac{G_{max}}{G_{min}}$$

Usually expressed in dB:

$$\mathrm{Dynamic}\mathrm{Range}=20\times \mathrm{lg}\frac{G_{max}}{G_{min}}\;$$

#### 2.4.4. Multilevel Conductances

For ferroelectric transistors: the gate voltage controls the ferroelectric domains flipped in the ferroelectric film to obtain different residual polarization strengths; i.e., different gate voltages correspond to different residual polarization strengths. The residual polarization state regulates the channel conductance state, and since the residual polarization state determines the threshold voltage of the transistor, so the number of carriers in channel varies with the residual polarization state, and the drain current is a function of the gate voltage. In ferroelectric transistors with linear symmetric conductance variations, either potentiation or depression, correspond to different conductance states by modulating different residual polarizations [61].

#### 2.4.5. Linearity

The process of synaptic weight increasing or decreasing under different stimulus is called long-term potentiation and long-term depression effect. In the face of the continuous change of synaptic weight, the parameter value of linearity is used to measure whether the synaptic weight linearly increases or decreases, and whether the weight in the two processes is symmetrical. Most of the synaptic devices actually manufactured at this stage rapidly increase at the early stage of enhancement, and the weight of depression rapidly decreases at the later stage. How to use appropriate formulas to fit this state is an important issue for researchers to consider. Seo proposed the following formula to fit the LTP/LTD characteristic curve of the artificial synapse; where *G_n_* represents the conductance value of the synaptic device at the nth stimulation, *G_n_*_+1_ represents the conductance value of the next pulse, *G_max_* and *G_min_* represent the maximum and minimum conductance values in the two process, respectively, and parameter *α* and parameter *β* represent the nonlinearity of the conductivity interval update [71,84,85].

$$G_{n+1}=G_{n}+∆G_{p}=G_{n}+\alpha_{p}e^{-\beta_{p}[(G_{n}-G_{min}/\left(G_{max}-G_{min)}\right]}$$

$$G_{n+1}=G_{n}+∆G_{D}=G_{n}-\alpha_{D}e^{-\beta_{D}[(G_{max}-G_{n}/\left(G_{max}-G_{min)}\right]}$$

## 3. Memory Advances with 2D Materials Heterostructure Devices for Constructing Neuromorphic Systems

Researchers have investigated 2D materials for their potential applications and numerous viewpoints, including optical components, quantum devices, and electronic devices. In recent years, two-dimensional materials and their heterogeneous architectures have demonstrated broadband optical response and high optical responsiveness, which are characterized by rapid switching, multiple data storage, and extensive on/off comparisons in memory [13,39]. In addition, the ultra-thin body thickness and low-temperature transfer enable the non-uniform integration of 2D materials with other material systems. Emerging electronic product categories, such as portable electronics, biomedical electronics, three-dimensional storage, and ultra-low power consumption, place a premium on the scalability of devices. Furthermore, 2D materials have enabled the development of a new scaling technology for devices. Over the past two decades, scientists have constructed a variety of heterogeneous structures based on popular two-dimensional materials such as graphene, molybdenum disulfide, hexagonal boron nitride (h-BN), etc. The advantages of a high switching ratio and a lengthy retention duration are utilized by the gadgets. These devices exhibit substantial neuromorphic computing application potential [37]. Due to charge-trapping carriers in the original or fabricated flaws at the interface that modify channel resistance, 2D-material-based memtransistors are sensitive to environmental variables. In memory devices, the charge-trapping technique typically results in relatively brief retention durations. Neuromorphic devices obtained by combining different types of 2D materials with other semiconductor materials to construct heterogeneous have greater potential and superior properties, such as light-sensitive range, environmental stability and responsiveness, non-volatility, data storage capacity, and synaptic properties [85,86].

**Figure 4:**
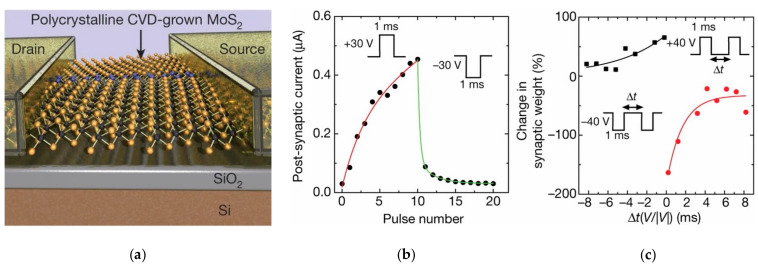
A memtransistor with a structure of SiO_2_/MoS_2_. (**a**) Schematic diagram of a SiO_2_/MoS_2_ memtransistor [87]; (**b**) Post-synaptic current changes with the +30 V and −30 V pulse number, showing long-term potentiation and depression. Under the continuous modulation of positive pulse, the post-synaptic current is enhanced continuously, showing a long-term potentiation effect, which can be well fitted by the red curve. The process of decreasing post-synaptic current can be well fitted with green curve under negative pulse voltage modulation [87]; (**c**) SiO_2_/MoS_2_ memtransistor simulates the spike-timing-dependent plasticity under the +40 V and −40 V pulse number. The curves in different colors represent the fitting of synaptic weight changes under different modulation modes [87]. Copyright © 2018, Macmillan Publishers Limited, part of Springer Nature. All rights reserved.

**Figure 5:**
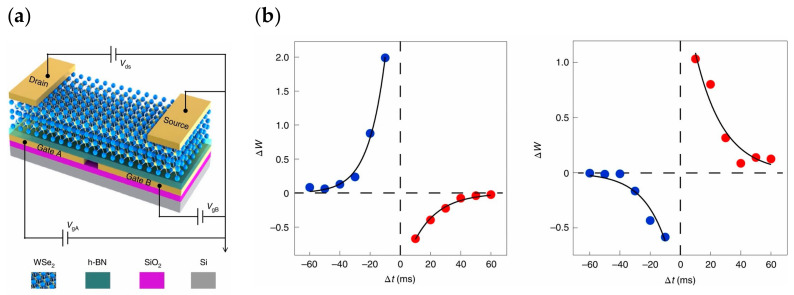
Bipolar field effect transistor with a structure of h-BN/WSe_2_. (**a**) Schematic diagram of h-BN/WSe_2_ bipolar field effect transistor [89]; (**b**) Simulation of STDP characteristics by bipolar FET, which shows two different types of synaptic plasticity learning rules. In the left figure, the blue curve fits under the condition of increasing synaptic weight, while the red curve fits under the condition of decreasing synaptic weight, both of which reflect the regulation mechanism of synaptic weight changing with pulse interval. The different color curves in the right figure also fit the changes in synaptic weight, but under different environmental conditions, the device will show different regulatory mechanismsIn the left figure, the blue curve fits under the condition of increasing synaptic weight, while the red curve fits under the condition of decreasing synaptic weight, both of which reflect the regulation mechanism of synaptic weight changing with pulse interval. The different color curves in the right figure also fit the changes in synaptic weight, but under different environmental conditions, the device will show different regulatory mechanisms [89]. Copyright © 2020, Chen Pan et al., under exclusive license to Springer Nature Limited.

**Figure 6:**
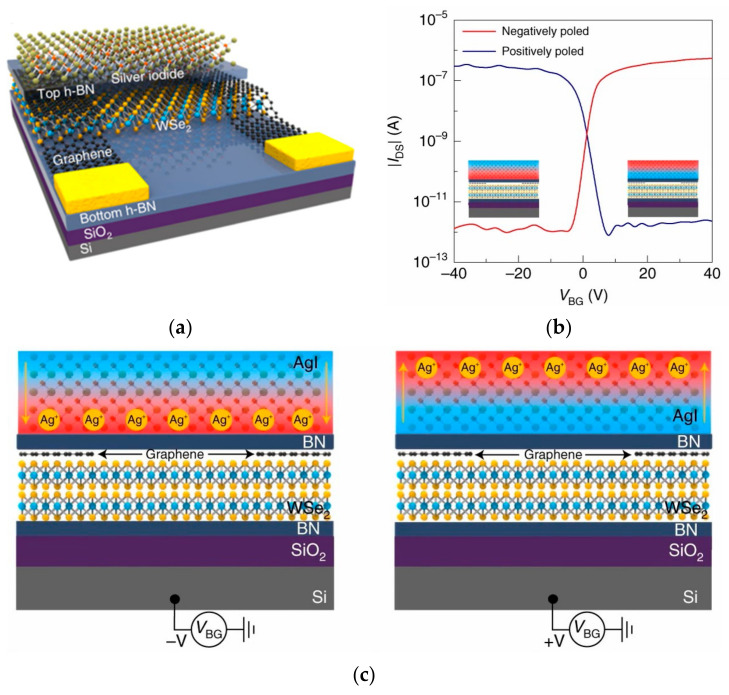
(**a**) Ion-doped field effect transistors [91]; (**b**) Device conducts bipolarly through phase transition [91]; (**c**) Ionic phase transition in silver iodide induces switchable ion doping [91]. Copyright © 2020, Sung-Joon Lee et al., under exclusive license to Springer Nature Limited.

Synapses are the fundamental elements of human neural networks, and the release of neurotransmitters in presynaptic neurons regulates synaptic signaling. When neuro-transmitters diffuse through the synaptic gap and dock with receptors in postsynaptic neurons, electrical stimulation generates a response in the postsynaptic nerve cell and triggers a postsynaptic current (PSC), allowing information transfer from presynaptic to postsynaptic neurons, where conductance (synaptic weight) regulation is referred to as synaptic plasticity [61]. In recent years, heterostructures have been adopted by researchers to build artificial synaptic devices. In 2017, Sangwan reported the realization of multi-terminal memristor transistors by stacking MoS_2_ on SiO_2_ in a related compatible process (Figure 4a). The device exhibits a superior switching ratio, as well as cycling durability and stability. It also possesses good emulation of learning behaviors, such as LTP/LTD and STDP in biological synaptic properties (Figure 4b,c). People can operate the device by combining CMOS transistors with it to select the desired individual state [87]. In 2020, Cho et al. reported a NbSe_2_/WSe_2_/Nb_2_O_5_ heterostructure constructed on SiO_2_/p^+^Si substrate to simulate a novel neuromorphic synapse, which can provide excellent transistor switching characteristics with some properties of two-dimensional materials and a good bonding mechanism [88]. In 2020, Pan et al. constructed a bipolar field effect transistor combining homogeneous and heterogeneous by stacking multiple layers of WSe_2_ on h-BN (Figure 5a). The use of WSe_2_ as a channel material allowed it to exhibit bipolar tuning, and using the drain voltage polarity of the device as a control part, reconfigurable digital logic functions were achieved by programming different combinations of input signals; at the same time, reconfigurable STDP and pulse-tunable synaptic potentiation or depression could be achieved with the help of a circuit composed of three devices, significantly reducing circuit complexity (Figure 5b). It shows great promise for the future use of this device for implementing reconfigurable multifunctional logic and neuromorphic systems [89]. In two-dimensional-material-related devices, the migration and redistribution of ions can cause local bias electric fields at the interface between the two-dimensional material and electrode contacts, resulting in a reduced dynamic range of conductance and linearity of the change curve of the device. To address this problem, in 2021, the ACS-related journal reported the development of neuromorphic electronic synapses with sulfur anion reservoirs by Song Hao et al. By stacking MoS_2_ and WO_3_ layers to form a heterogeneous structure and acting as an anion storage pool with the help of the WO_3_ layer, the problems were effectively solved. The prepared device possesses high stimulus responsiveness and achieves nearly linear conductance changes and up to 130 conductance states in the long-range regulation, and the artificial neural network built with the device achieves a 93.2% recognition rate in a dataset [90]. In 2020, Nature Electronic reported the important results of Lee et al. who successfully achieved doping in two-dimensional materials. Two-dimensional semiconductors have an atomic-level thickness, which facilitates the construction of next-generation electronic devices at the nano level. However, controlling the conductive polarity of 2D materials by doping is difficult due to the limited physical space between atomic lattices. Based on a solid-state ion doping approach, Lee et al. used superionic phase transitions in silver iodide to induce switchable ion doping and constructed related devices by stacking multilayers of tungsten diselenide (WSe_2_) (Figure 6a–c), successfully achieving reconfigurable devices with carrier-type transistors and diodes with switchable polarity. In addition to this, the integration of ion-modulated transport with 2D semiconductors is highly likely to facilitate the development of electronic devices by effectively coupling electron transport with ion transport, resulting in novel devices that integrate both functions in unconventional computing, information storage, and advanced solid-state neuromorphic circuits [91].

**Figure 7:**
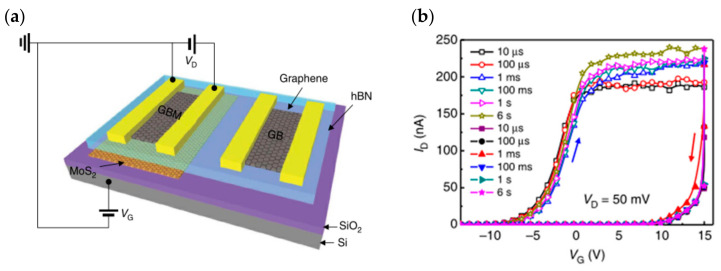
(**a**) Schematic diagram of ultra-thin heterostructure memory devices [92]; (**b**) Device storage window regulation. The transfer characteristic curve of the device is measured by applying pulse on the gate, in which the different color curve represents different pulse width [92]. Copyright © 2013, Nature Publishing Group, a division of Macmillan Publishers Limited. All Rights Reserved.

**Figure 8:**
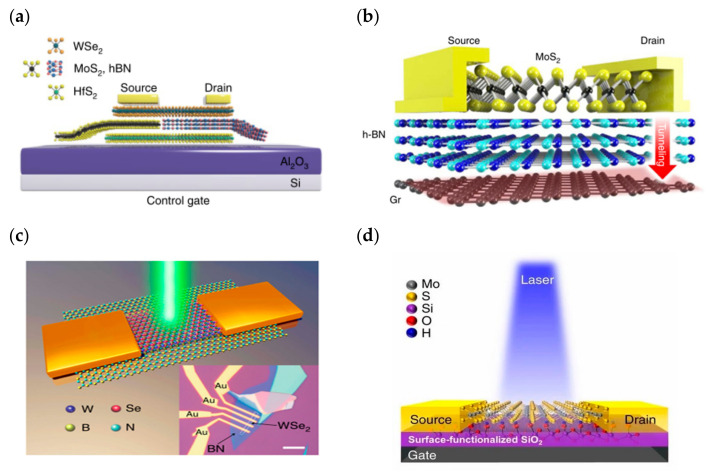
(**a**) Schematic diagram of a quasi-non-volatile floating gate memory device [93]. Copyright © 2018, Chunsen Liu et al.; (**b**) Graphene/h-BN/MoS2 vertical stacking to form floating grid memory [94], Copyright © 2016, Quoc An Vu et al.; (**c**) Schematic of WSe2/BN multi-bit non-volatile optoelectronic memory [66], Copyright © 2018, Du Xiang et al.; (**d**) Schematic diagram of MoS2 optoelectronic memory device [95]. Copyright © 2017, Juwon Lee et al.

While purely simulating biological synaptic properties, some researchers have taken the memory perspective to build new memory devices through heterogeneous structures, which usually have low power consumption, fast programming operation, and multi-state storage, and also have the potential for neuromorphic computing. In 2013, Choi, Lee et al. stacked graphene/h-BN/MoS_2_ to achieve ultrathin heterostructure memory devices (Figure 7a), in which graphene and MoS_2_ are used as channel and charge capture layers, and carriers reach the floating gate position through the tunneling layer h-BN to control the carrier transport in the channel. By changing the thickness of the two-dimensional materials or changing the stacking order, the device storage window size and conducting polarity can be controlled (Figure 7b); finally, the device exhibits a high current switching ratio, high mobility, and good stability, etc. [92]. In 2018, Zhou Peng’s team constructed a quasi-nonvolatile floating gate memory device using two-dimensional materials such as WSe_2_, MoS_2_, h-BN, and HfS_2_ with a special stacking structure (Figure 8a). The device construction process is fully compatible with silicon-based technology, which facilitates the construction of an interoperable bridge between volatile and non-volatile storage, reduces the power consumption for high-speed frequent erasing and reading, and enables the construction of high-speed and low power consumption memories [93]. In 2016, Nguyen, Kim et al. reported a floating-gate memory made of graphene/h-BN/MoS_2_ vertically stacked (Figure 8b). A similar tunneling charge capture mechanism was used to charge and discharge the floating gate, and the final device showed a current switching ratio of up to 10^9^ and an off-state current down to an order of 10^−14^; the device also had excellent stretching properties, revealing its great promise for flexible wearable devices [94]. In 2018, a multi-bit non-volatile optoelectronic memory based on a single layer of tungsten diselenide and a small number of hexagonal boron nitride heterostructures was reported by a related research team at the National University of Singapore (Figure 8c). The tungsten diselenide/boron nitride memory showed a memory switching ratio of approximately 1.1 × 10^6^, guaranteeing more than 128 (7 bits) different storage states with a retention time of more than 4.5 × 10^4^ s [66]. In 2017, Juwon and Sangyeon et al. reported the concept of a monolayer MoS_2_ optoelectronic memory device (Figure 8d), which operates through the monolayer/dielectric interface functionalization using an artificially structured charge trap layer to induce local electron capture and release. The built device has excellent photoresponsivity memory characteristics with a large linear dynamic range of ~4700 (73.4 dB), a low turn-off current (<4 pA), and a storage lifetime of more than 10^4^ s. In addition, multi-stage detection of up to eight optical states was successfully demonstrated [95].

**Figure 9:**
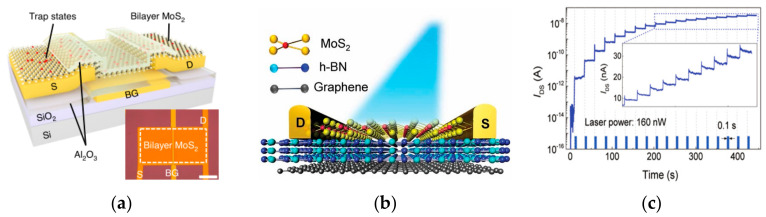
(**a**) Double−layer MoS2-built vision sensor devices [96]. Copyright © 2022, Fuyou Liao et al., under exclusive license to Springer Nature Limited; (**b**) Multi−state non−volatile optical memory devices based on MoS_2_/h−BN/graphene heterostructures [97]; (**c**) Formation of multi−level conductivity states with the continued light pulse for MoS_2_/h-BN/graphene heterostructures [97]. © 2018 WILEY−VCH Verlag GmbH & Co. KGaA, Weinheim.

**Figure 10:**
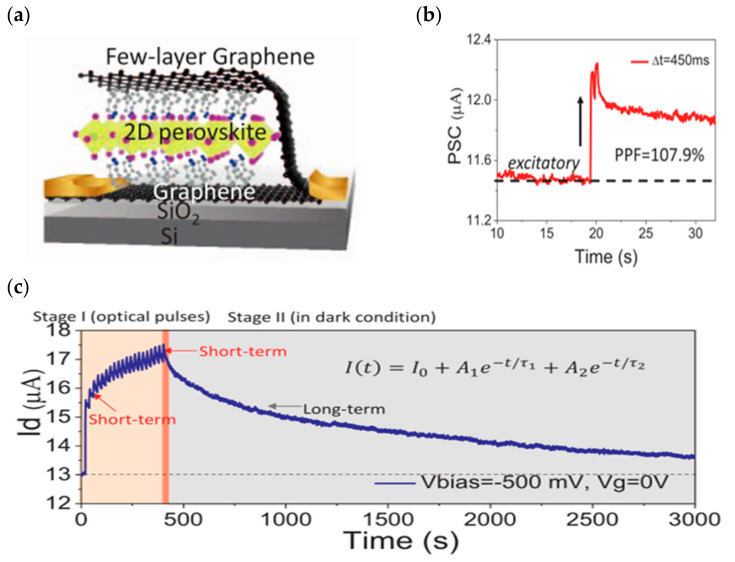
Artificial optical synapses. (**a**) Schematic diagram of graphene/2D perovskite/graphene sandwich structure for constructing ultra-sensitive artificial optical synapses [98]; (**b**) Artificial optical synaptic light control PPF test [98]; (**c**) Artificial optical synaptic short-range plasticity and long-range plasticity testing result [98]. Copyright © 2018, IEEE.

**Figure 11:**
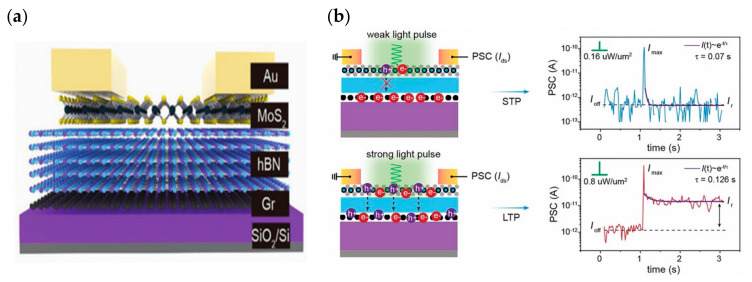
(**a**) Schematic diagram of MoS_2_/h−BN/Gr architecture optical artificial synapse [99]; (**b**) Optical artificial synaptic simulation of biological properties. Under the weak light pulse stimulation, the device shows short range plasticity, and the synaptic current returned to the initial state after the removal of the light pulse. The device shows long-term plasticity under the stimulation of strong light pulse, and the post−synaptic current remained at a high level after the removal of light pulse [99]. © 2022 Wiley−VCH GmbH.

Based on the good optical properties of some two-dimensional materials, some researchers have developed optoelectronic modulation of FETs, which enables the simulation of synaptic properties through optical/electrical synergistic control. In 2022, Ahn and Chai et al. reported their vision sensor devices constructed using a bilayer MoS_2_ on a high-K dielectric (Figure 9a). By introducing trap states on the MoS_2_ surface and using the trap to store light information, they were able to dynamically modulate the characteristic curve of the device under different lighting conditions. The device shows a dynamic sensing range of up to 199 dB [96]. In 2018, to address a series of issues such as high programming voltage, high static power consumption, and difficult integration in the development of three-terminal optoelectronic memory devices, Tran et al. developed a multilevel nonvolatile floating-gate optical memory device based on a MoS_2_/h-BN/graphene heterostructure (Figure 9b). The device exhibited a current switching ratio of up to 10^6^, the turn-off current could be maintained at a very low level of 10^−14^ A, and the endurance cycle degree and retention time reached 10^4^ cycles and 3.6 × 10^4^ s. The channel could be effectively modulated by controlling the migration of electrons in the graphene layer through an applied photoelectric stimulus (Figure 9c), thus realizing an optical memory device with a multilevel conductive state [97]. In 2018, Tian et al. innovatively employed a distributed architecture by stacking graphene/2D perovskite/graphene interlayer structures on SiO_2_ substrates (Figure 10a) to achieve an optical memory device that has 730 A/W responsiveness and a 74-day retention time. In addition to its good optical response, the device is able to achieve reconfigurable biological synaptic properties with the help of optical modulation (Figure 10b,c) and can achieve a good degree of simulation of learning behaviors, such as PPF and STDP [98]. In 2022, Yang et al. built a novel optoelectronic artificial synapse based on vdw heterostructures (Figure 11a), which was made by vertically stacking MoS_2_/h-BN/graphene on a Si/SiO_2_ substrate, and by artificially modulating the energy bands of the structured material. The device demonstrated positive (PPC) and negative photoconductance NPC optoelectronic coupling modes; based on this mode, the authors successfully constructed a variety of digital logic gates with reconfigurable capabilities. In addition, the device allows for the conductance modulation of the photoelectric dual mode (Figure 11b), and successfully simulates the biological synaptic properties such as STP and LTP/LTD [99].

**Figure 12:**
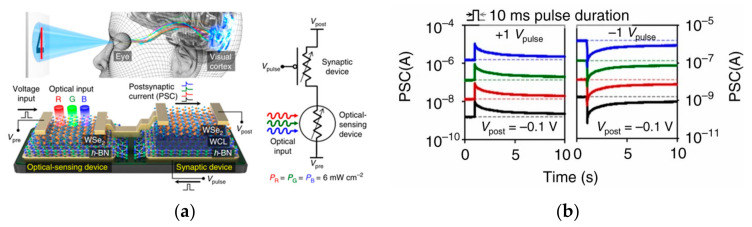
(**a**) Schematic diagram of the h−BN/WSe_2_ heterogeneous structure vision sensing device [64]; (**b**) Electric pulses of different polarity are applied to the device under different light conditions to simulate the excitation and inhibition of synapses. The black curve represents the dark environment, and the remaining curves represent the corresponding color light test environment [64]. Copyright © 2018, Seunghwan Seo et al.

**Figure 13:**
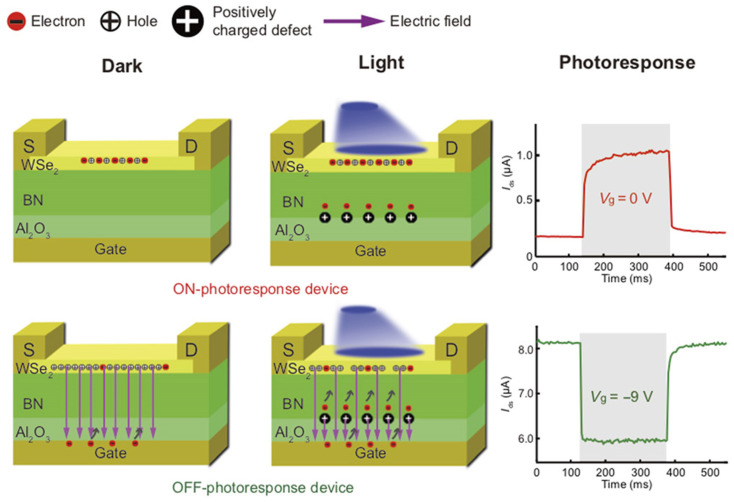
WSe_2_/h-BN/Al_2_O_3_ heterostructure for bipolar cell simulation [63]. Copyright © 2020 Chen-Yu Wang et al., some rights reserved; exclusive license American Association for the Advancement of Science.

**Figure 14:**
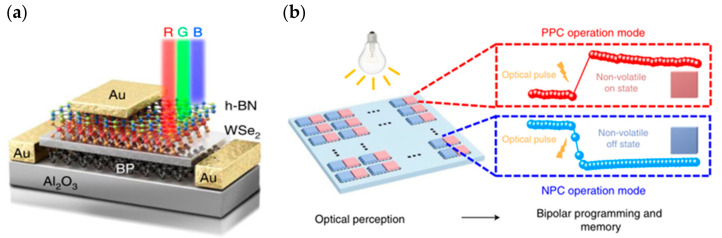
BP/Al_2_O_3_/WSe_2_/h-BN heterostructures [62]. (**a**) BP/Al_2_O_3_/WSe_2_/h-BN heterostructures simulate retina-related structures [62]; (**b**) PPC/NPC simulation of BP/Al_2_O_3_/WSe_2_/h-BN heterostructures [62]. Copyright © 2021, Zhenhan Zhang et al., under exclusive license to Springer Nature Limited.

**Figure 15:**
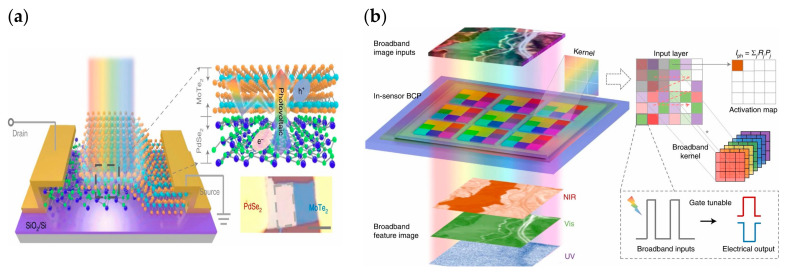
PdSe_2_/MoTe_2_ vdw heterostructure. (**a**) Schematic of broadband convolution processing sensor for PdSe_2_/MoTe_2_ vdw heterostructure [65]; (**b**) Wideband convolution processing sensor implements different types of convolution processing. The gate pluse V_g_ is used to adjust the optical responsiveness R_j_, the incident light represents the input layer, the optical power P_j_ represents the pixel value, and the final result of the convolutional operation represents the output optical current. Each pixel can achieve weight adjustment and can achieve positive and negative light response [65]. Copyright © 2022, Lejing Pi et al. under exclusive license to Springer Nature Limited.

Despite the bimodal modulation of device optoelectronics, researchers have expanded the practical application scenarios of neuromorphic devices. In 2018, Choi et al. from Korea presented their research results on artificial visual synapses for color mixed-mode recognition by implementing both synaptic and optical sensing functions on the h-BN/WSe_2_ heterogeneous structure (Figure 12a). An optic nerve synaptic device was constructed by this device to demonstrate a visual sensing device with synaptic and optical sensing functions; the device exhibited different synaptic behaviors such as LTP/LTD and STDP depending on the light conditions (Figure 12b); also, a nearly linear weight update trajectory was demonstrated in terms of synaptic plasticity, and the device was able to provide a large number of stable conductance states in operation (each state’s variation of less than 1%) [64]. In 2020, Feng Miao’s team also demonstrated its simulation of bipolar cells and photoreceptors in living organisms in Science Advances. The device uses a WSe_2_/h-BN/Al_2_O_3_ heterostructure (Figure 13), which vertically integrates photoreceptors and bipolar cells through a heterostructure that is simple and compact compared to the complex structure of a silicon retina. The authors relied on this device to build pixel arrays and achieve reconfigurable artificial vision sensors by adjusting the gate voltage of each pixel for simultaneous image sensing and processing; in iterative training for image recognition, an accuracy of 100% was achieved in less than 10 cycles [63]. In 2021, Peng Zhou’s team created a heterostructure device using two-dimensional materials, h-BN and WSe_2_, to replicate the structural functions of the retina, and ultimately achieved efficient dynamic monitoring detection. Figure 14a is a diagrammatic representation of the BP/Al_2_O_3_/WSe_2_/h-BN heterostructure device structure. The authors used the device to construct the associated circuitry for an integrated sensory-storage and computational biorational analog device that senses optical stimulus, collects and converts signals to simulate an image perceptron, and permits stimulation by programmable electrical and optical pulses to generate a non-volatile positive photocurrent (PPC) and a negative photocurrent (NPC) (Figure 14b) [62]. Broadband convolution processing is crucial for high-precision image recognition; however, it is challenging to implement broadband convolution processing for sensors using conventional CMOS technology. Based on this problem, the research team of Zhou published their research results regarding broadband image sensing and convolution processing with a vdw heterostructure device this year. In the report, the authors used PdSe_2_/MoTe_2_ to build a vdw heterostructure broadband convolution sensor (Figure 15a). The heterostructure device has a gate-tunable positive and negative optical response, as well as a broadband linear gate-correlated optical response, which allows for different types of convolution processing of remote sensing images (Figure 15b). The broadband convolutional processing within this sensor improves the recognition accuracy of multi-band images compared to conventional single-band convolutional neural networks [65].

## 4. Ferroelectric Field Effect Transistors for Building Artificial Synaptic Elements

Ferroelectricity is a physical property shown by several dielectric substances. Many ferroelectric compounds exist in nature and are collectively known as ferroelectrics. The presence of spontaneous polarization and the reversal of polarization direction in response to a change in the applied electric field are the two most important features of ferroelectrics [46,49]. The introduction of perovskite oxide ferroelectric materials in the 19th century, the development and perfection of the physical theory relating to ferroelectricity in the 20th century, and the emergence of nanomaterials in the middle of the 1980s all led to a rapid improvement in the ferroelectric materials preparation technology level. The novel concepts of nanoscale ferroelectricity, ferroelectric thin film devices, ferroelectric thin film batteries, etc., have attracted the attention of scientists to a great degree. Moreover, the requirement for miniaturization, high-density, and low-cost electronic devices has increased as a result of the ongoing progress of microelectronics technology, as shown by integrated circuits. Conventional ferroelectric materials have collided and combined with semiconductor systems, ushering in the era of integrated ferroelectrics in ferroelectricity research [51,71].

Common ferroelectric materials can be divided into three categories: inorganic ferroelectrics, organic ferroelectrics, and two-dimensional ferroelectrics, with inorganic ferroelectrics being the most widely used at this time, especially in the memory field; with organic ferroelectrics being more applicable to certain specific scenarios and two-dimensional ferroelectrics having superior performance and the potential to shine in the development of integrated circuits in the future. Regarding the application of ferroelectric materials in the field of new devices for neuromorphic computing, there have been more reports on two-terminal devices, which are typically presented as a simple metal/insulator (ferroelectric dielectric)/metal sandwich structure and can realize the resistive behavior of the device and the simulation of synaptic properties via ferroelectric flipping. Based on a simple sandwich structure, these devices are highly scalable and have great potential for high-density cross-array integration, but they have major shortcomings in the precise regulation of linear gradient conductance [100]; as a result, multi-terminal transistors utilizing ferroelectric materials as gate dielectrics have become a research target for researchers to advance this field.

### 4.1. Inorganic Ferroelectric Gate Field Effect Transistors for Building Artificial Synapses

**Figure 16:**
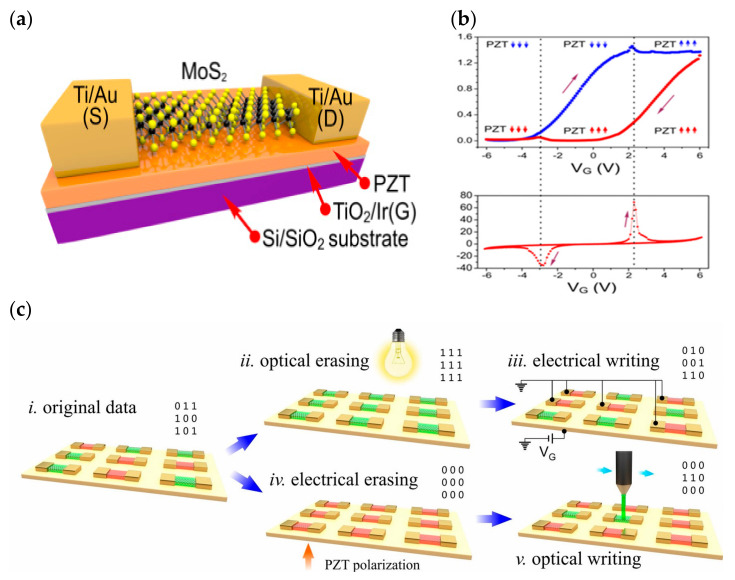
(**a**) Schematic diagram of PZT gate dielectric device structure [69]; (**b**) Formation of hysteresis window [69]; (**c**) Device optical/electrical erase and write operation demonstration [69]. Copyright © 2015, American Chemical Society.

**Figure 17:**
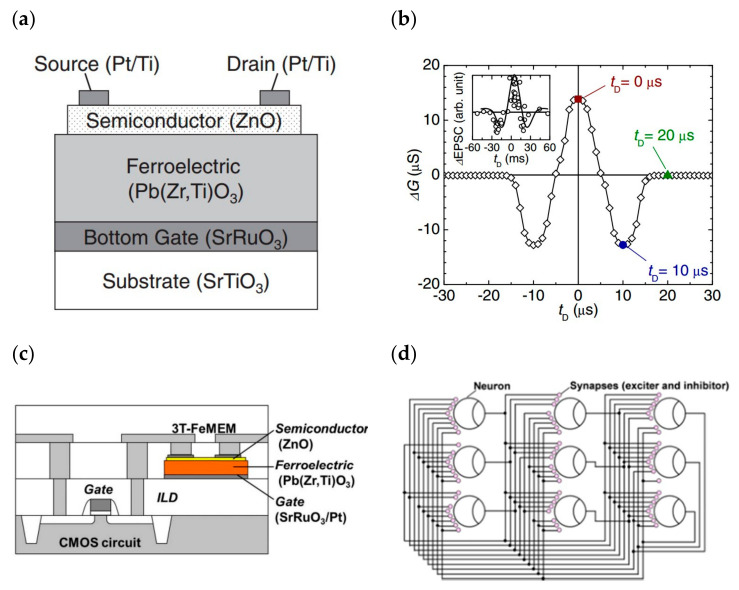
(**a**) Schematic diagram of the artificial synapse of ZnO/PZT structure [101]; (**b**) The device successfully implements the simulation of one of the STDP modes [101]. © The Japan Society of Applied Physics. Reproduced by permission of IOP Publishing Ltd. All rights reserved; (**c**) Construction of ZnO/PZT structured artificial synapses on CMOS [73]; (**d**) Neural networks constructed by artificial synapses [73]. Copyright © 2014, IEEE.

The synaptic weight change of ferroelectric artificial synapses arises from the multilevel nonvolatile polarization modulation of ferroelectrics, which, when matched with suitable electrode materials or buffer layers, can significantly increase their cyclic durability [34]. In ferroelectric neuromorphic transistors, the polarization state of the ferroelectric layer is controlled by gate pulses, and the source-drain current is used as an indirect reflection of its polarization state. Additionally, successive pulses are used to modulate the incremental or decremental ferroelectric polarization in order to realize the multilevel conductivity state. Early researchers focused on the application of FeFET in high-performance memory, which stores information through the hysteresis window formed by ferroelectric polarization, and used inorganic ferroelectric materials as gate dielectrics to regulate transistors, such as chalcogenide (PZT), hafnium oxide, hafnium oxide-doped materials, etc. In 2015, U.S. researchers Alexander Sinitskii et al. exhibited an optoelectronic memory device based on PZT and MoS_2_ (Figure 16a). The device employs the ferroelectric material PZT as the gate dielectric, and a monolayer molybdenum disulfide (MoS_2_)-based field-effect transistor was built and tested on a substrate to confirm that the device has a large hysteresis window (Figure 16b). The device enables both optical/electrical write and erase operations (Figure 16c), making it easier to use than traditional similar devices; the threshold voltage drift phenomenon caused by ferroelectric polarization confirms the device’s ability to operate in the neuromorphic field [69]. In 2013, Yu Nishitani effectively simulated the biological synaptic learning function with the ZnO channel material and the PZT ferroelectric gate dielectric-prepared FeFET (Figure 17a). By altering the channel conductance to reflect the mass of the synaptic basic unit, it was possible to simulate features such as STDP in the plasticity of artificial synaptic devices (Figure 17b) [101]. The following year, Yu Nishitani et al. took the previous year’s device development results and expanded them at the device application level. Using several FeFETs as leaky integrate fired (LIF) neural network models in conjunction with CMOS technology, a PZT gate dielectric FeFET on a CMOS circuit base was successfully stacked (Figure 17c). The constructed neural network consists of nine neurons and one hundred and forty-four synapses (Figure 17d), which, with the aid of nonvolatile continuous linear conductance modulation of FeFET and STDP learning rules, forms a correlation learning matrix capable of recalling the initial pattern by auto-learning when presented with incomplete multiple shaded pattern inputs. In addition, this FET is applicable to different types of neural network models, demonstrating the enormous potential of this artificial synapse for future applications in huge neuromorphic circuits [73].

**Figure 18:**
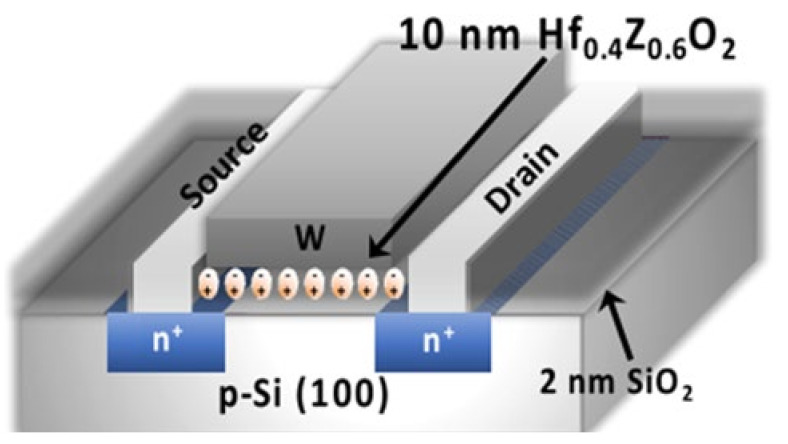
Schematic diagram of the new FeFET with HZO gate medium [72]. Copyright © 2018, IEEE.

FeFETs with inorganic chalcogenide gate dielectrics have significant limitations; because materials such as PZT are more complex and riskier to produce, and because the ferroelectricity of chalcogenide materials disappears at a certain thickness, it limits the possibility of continuous miniaturization of the characteristic size of these devices. The reported discovery of ferroelectricity in hafnium oxide has shed new light on the evolution of this subject in this context. The ferroelectric properties of hafnium oxide can be effectively improved by doping it with other elements (Si, Zr, etc.); hafnium oxide as a high K material has been used in relevant CMOS integrated circuit processes, and its development process is mature and compatible with existing CMOS semiconductor processes. Based on the above kinds of conditions, more reports on hafnium oxide or doped hafnium oxide dielectric transistors have also appeared in recent years. In 2018, Matthew Jerry et al. published their research regarding the fabrication process, parameter performance characterization, and analytical modeling of ferroelectric field-effect transistors (Figure 18). The results revealed the capacity to cause a subthreshold swing (SS) 2.3 kT/q, near-zero hysteresis negative drain-induced potential barrier reduction, and negative differential resistance in ferroelectric dielectric with the aid of an internal polarization flip. In addition, the causes of V_t_ drift in FeFETs were identified and future development guidelines for FeFETs were proposed [72].

**Figure 19:**
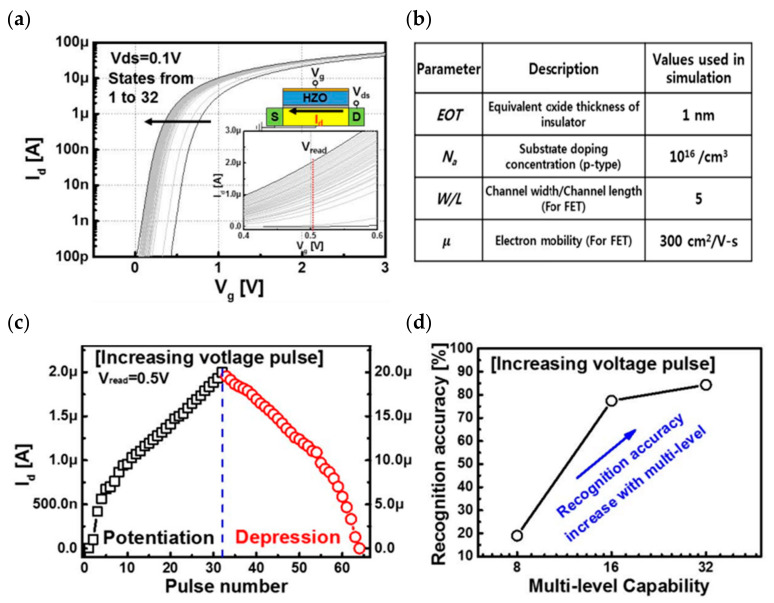
HZO-based ferroelectric synapse device. (**a**) Simulation results of the transfer curve for different polarization states [71]; (**b**) The parameters in simulation [71]; (**c**) 32 levels for potentiation and depression [71]; (**d**) Pattern recognition accuracy increases with multi-level capability [71]. Copyright © 2017, IEEE.

**Figure 20:**
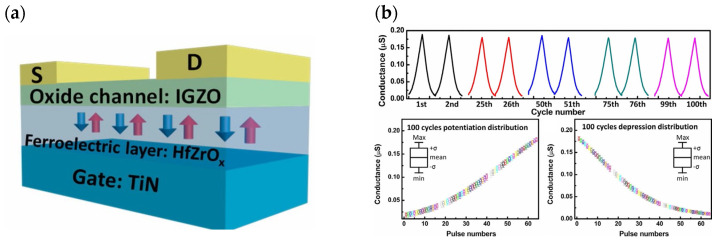
(**a**) Schematic of FeFET with IGZO channel [74]; (**b**) Experimental measurement result of LTP/LTD effects through IGZO channel devices. In the figure above, different colors represent different number of long range modulation cycles with an interval of 25 cycles. It can be seen that pulse stimulation can still achieve good regulation of device conductance after 100 cycles. The different colors in the figure below represent the conductance state of the device when different pulses are applied [74]. Copyright © 2019, American Chemical Society.

**Figure 21:**
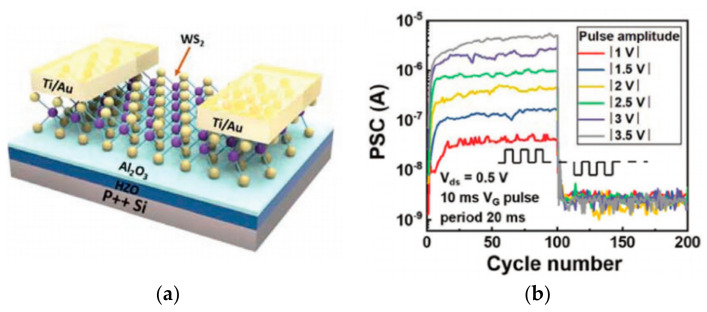
(**a**) Schematic of a device with WS_2_/HZO structure [55]; (**b**) Experiment test result of the LTP/LTD effect with different amplitude voltages on it [55]. © 2020 WILEY−VCH Verlag GmbH & Co. KGaA, Weinheim.

In addition to the research on high-performance FeFETs, reports of the application of this type of transistor to build artificial synapses have also emerged. In 2017, Seungyeol Oh proposed a novel type of HZO-based ferroelectric synapse device (Figure 19). The researchers successfully identified 32 ferroelectric residual polarization states possessed by the ferroelectric dielectric by employing various pulse test methodologies, and subsequently exploited this condition to develop a device correlation model. Si was still used as the channel material in the model, and the final simulation results demonstrated that the device was able to achieve superior LTP/LTD modulation effects, as well as strong symmetry of conductance changes and high linearity in the enhancement and suppression effects; the application to the MNIST dataset revealed an accuracy of 84%, indicating that the HZO-based synaptic device has potential for future applications in high-density neuromorphic systems [71]. In the same year, H. Mulaosmanovic et al. successfully produced a single ferroelectric artificial synapse utilizing 28 nm HKMG technology and a TiN/Si, HfO_2_/SiON/Si stack structure (where HfO_2_ is the ferroelectric gate dielectric and SiON is the channel material). With the aid of nonvolatile ferroelectric regulation, a continuous change in channel conductance could be achieved in order to simulate the LTP/LTD effect; additionally, by controlling the time interval between pre-pulses and post-pulses, a change in channel conductance could be observed, thereby enabling the simulation of the STDP learning mechanism [102]. In 2019, Min-Kyu Kim et al. effectively reproduced some synaptic properties using a nanoscale thickness of an HZO ferroelectric thin film transistor (FeTFT) constructed with ferroelectric gate dielectric material and oxide semiconductor channel material (IGZO) (Figure 20a). The FeTFT was able to achieve potentiation and depression behavior with a linearity of −0.8028/−0.6979 in long-range modulation using ferroelectric polarization modulation (Figure 20b), while the ratio of maximum to minimum values in conductance modulation exceeded 14.4; the neuromorphic computational system constructed with this device was trained to achieve 91.1% accuracy in the recognition of handwritten digit sets [74]. In 2020, one year after Kim’s study was published, Ang’s team presented a new ferroelectric synaptic transistor based on the integration of two-dimensional WS_2_ and inorganic ferroelectric HZO (Figure 21a). The device construction procedure is fully compatible with existing semiconductor fabrication processes, and the ferroelectric layer’s stability is exceptional. The researchers studied the influence of the annealing temperature on the residual polarization strength of the ferroelectric HZO material, while the transistor was able to obtain a current switching ratio of up to 10^5^ based on the modification of the ferroelectric polarization direction. In addition, by applying pulsed stimulation at the gate control end, the ferroelectric synapse was able to imitate biological synaptic properties, such as EPSC and LTP/LTD (Figure 21b), demonstrating the device’s enormous potential for future neuromorphic engineering applications [55].

### 4.2. Organic Ferroelectric Materials for Building a Neuromorphic Synaptic

In addition to the inorganic class of ferroelectrics being used to build FETs, the organic class of ferroelectric materials also has some potential in building synaptic devices, such as some artificial synapses with the help of P(VDF-TrFE) as the gate medium [103]. In the production of organic ferroelectric FETs, PVDF films are frequently produced by spin coating on the appropriate dependent layers; similarly, to improve the ferroelectricity of PVDF films, annealing is required, albeit at a lower temperature (less than 200 °C) than for inorganic ferroelectric materials [104,105]. In particular, during the precipitation of organic ferroelectric materials, attention must be paid to the contact interface with channel materials and electrode materials, etc., as there are significant differences in their formation and structure compared to inorganic materials, and the compatibility of the process must be considered in the process that follows precipitation.

**Figure 22:**
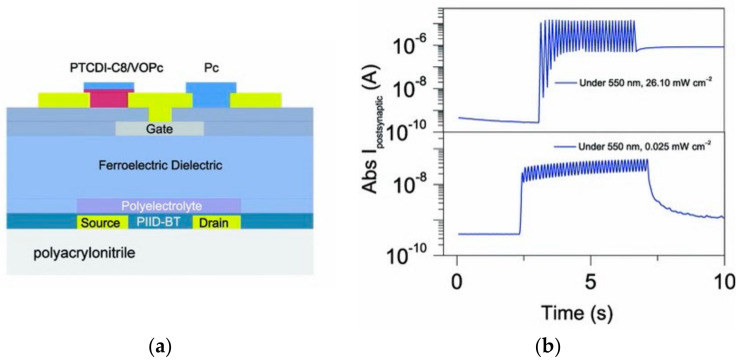
(**a**) P(VDF−TrFE)/P(VP−EDMAEMAES) gate−mediated artificial synapses [77]; (**b**) STP−LTP conversion through optical control [77]. © 2018 WILEY−VCH Verlag GmbH & Co. KGaA, Weinheim.

**Figure 23:**
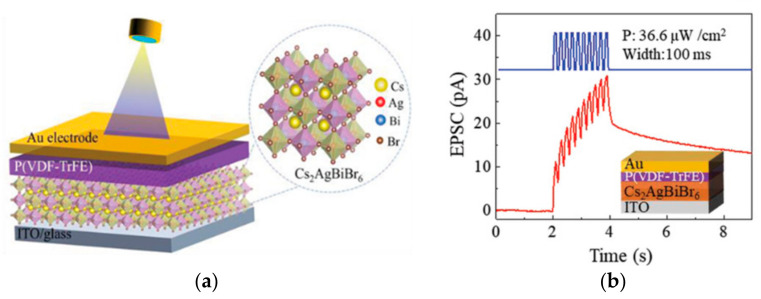
(**a**) Schematic diagram of the structure of two-terminal PVDF ferroelectric parts [106]; (**b**) EPSC simulation of two-terminal PVDF device [106]. © 2022 Jie Lao et al. Advanced Science published by Wiley−VCH GmbH.

**Figure 24:**
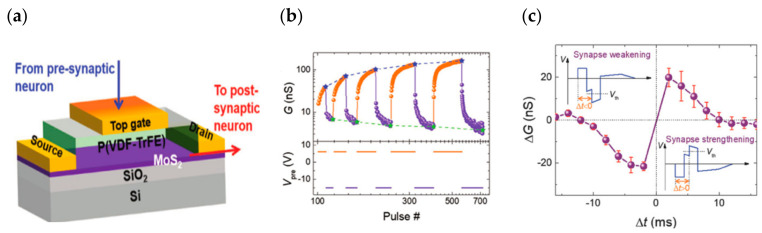
(**a**) Schematic diagram of PVDF−MoS_2_ structural device [107]; (**b**) Experiment simulation of LTP/LTD effects with different types of pulse number increases in PVDF−MoS_2_ structured devices. The orange curve shows the long range potentiation of the device under positive pulse voltage. The more the number of pulses, the higher the maximum conductivity state the device can achieve. The purple curve shows the long range depression under negative pulse voltage [107]; (**c**) Simulation of STDP learning mechanism by PVDF−MoS_2_ structured devices [107]. © 2018 WILEY−VCH Verlag GmbH & Co. KGaA, Weinheim.

In 2018, Hanlin Wang successfully fabricated a ferroelectric/electrochemical artificial synapse using the organic ferroelectric substance P(VDF-TrFE)/P(VP-EDMAEMAES) as the gate control medium (Figure 22a). The device achieves the simulation of synaptic behaviors such as STP and LTP through the degree of electrochemical doping by ferroelectric polarization, and integrates sensing and storage in a single device, while different forms of transitions such as STP can be achieved by applying pulses of amplitude or frequency to the gate (Figure 22b). This work extends the non-volatile retention period of the device to 10^4^ s, which is much longer than the minute-level retention time of other typical electrochemical transistors reported by prior studies. The neuromorphic visual perception system constructed using this device is able to transform light signals of varying frequencies and intensities into corresponding synaptic impulses, and the converted signals can differentiate between volatile and nonvolatile features [77]. The change in the polarization state of the ferroelectric body by the applied electric field affects its potential barrier distribution, and the average barrier height of the interlayer is typically used as a criterion to delineate the high/low resistance state of the device to achieve the data storage function; however, the change in the potential barrier under the applied electric field is not abrupt, it gradually changes with the change in the electric field, thereby requiring a different criterion to determine the high/low resistance. This indicates the possibility of creating neuromorphic blocking devices for two-terminal ferroelectric devices. In 2022, Bobo Tian’s team designed an ultra-low power machine vision sensor composed of a self-powered Au/P(VDF-TrFE)/Cs_2_AgBiBr_6_/ITO device (Figure 23a), which exhibited excellent bio-synaptic optoelectronic properties (Figure 23b), and was capable of performing both static and dynamic vision tasks with a 99% accuracy in applications of face classification and dynamic traffic flow recognition. The device is 99.97% and 100% accurate for face classification and dynamic traffic recognition [106]. Based on the successful preparation of the two-terminal synaptic device, the team further developed a low-power, highly robust three-terminal memtransistor with the help of ferroelectric polymer PVDF as the gate dielectric and MoS_2_ as the channel (Figure 24a). The device achieves quasi-continuous and precise conductance regulation with the help of ferroelectric polarization regulation, and successfully simulates synaptic characteristic behaviors such as LTP/LTD and STDP (Figure 24b,c). In addition, the device has high stability, very low power consumption, and a long lifetime. After extensive pulse tests, the switching ratio of the device still exceeds 10^4^, the power consumption per pulse test is less than 1 fJ, and the operating lifetime at specific frequencies exceeds 10 years; this demonstrates the great potential of the device for large-scale neuromorphic circuits [107].

### 4.3. Two-Dimensional Ferroelectric Materials Field-Effect Transistor Builds Neuromorphic Synapses

While traditional ferroelectric and two-dimensional materials are widely used to construct new high-performance field-effect transistors, researchers have progressively become aware of an emergent ferroelectric body. In_2_Se_3_ is a typical two-dimensional ferroelectric material with a direct band gap and a small effective mass of electrons; in 2014, a report on the preparation of In_2_Se_3_ transistors was published, but the performance of the transistors mentioned in this report was abysmal compared to the devices prepared by their counterparts, such as InSe [108]. It was not until 2017 that Wenjun Ding proved the existence of two-dimensional ferroelectric semiconductors for the first time at the theoretical level by demonstrating that monolayer In_2_Se_3_ has spontaneous ferroelectric polarization performance at room temperature through relevant theoretical calculations [109], which also unveiled a new dimension in the development of ferroelectric and two-dimensional materials.

**Figure 25:**
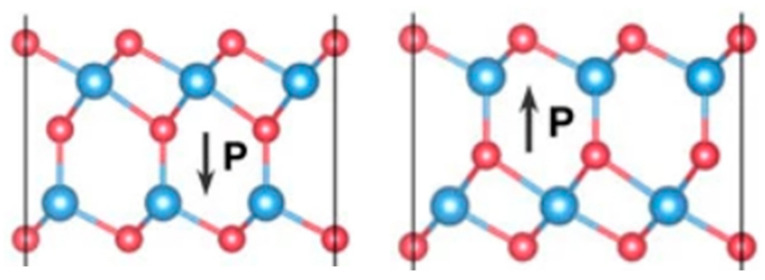
In_2_Se_3_ crystal structure in different polarization directions [109]. Copyright © 2017, Wenjun Ding et al.

**Figure 26:**
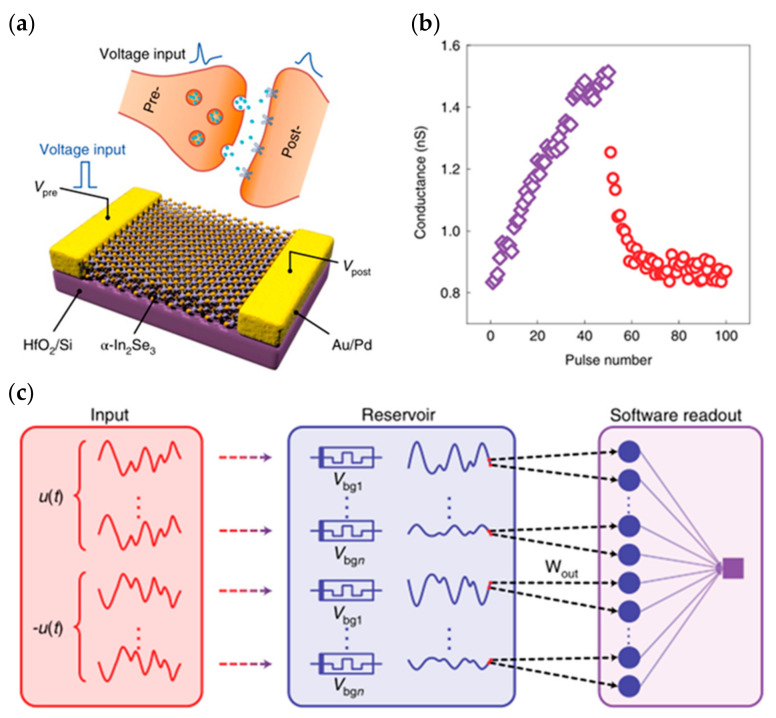
In_2_Se_3_ photoelectric synapse [76]. (**a**) Schematic diagram of In_2_Se_3_ photoelectric synapse [76]; (**b**) Photoelectric synapses simulated LTP/LTD performance [76]. (**c**) Schematic of a multiple-timescale RC system [76]. Copyright © 2022, Keqin Liu et al., under exclusive license to Springer Nature Limited.

Similar to conventional inorganic/organic ferroelectric materials, the 2D ferroelectric material In_2_Se_3_ can also form two distinct polarization directions in the presence of an external stimulus (Figure 25), and the modulation of a ferroelectric device is achieved by regulating the channel using the polarization effect [110]. The characteristic curve hysteresis phenomenon of 2D ferroelectric material transistors is caused by the internal polarization of the channel material, so it avoids the effects of depolarization and interfacial charge shielding, etc., and mitigates the issues of gate leakage and electrode contact. In 2020, Lin Wang et al. prepared a ferroelectric semiconductor field effect transistor (FeSFET) based on α-In_2_Se_3_. The researchers covered a 50-nm high K alumina dielectric with α-In_2_Se_3_ flakes, using Ti/Au as the source-drain electrode, and finally, covered with a 15-nm thick alumina passivation layer. By modulating different degrees of ferroelectric polarization, the device successfully simulated the biological synaptic properties of EPSC, IPSC, LTP/LTD, and STDP, and achieved a 91.9% pattern recognition rate in artificial neural networks, demonstrating the great potential of the device for neuromorphic circuit construction [111]. In 2022, Keqin Liu et al. also developed an optoelectronic synapse based on α-In_2_Se_3_ that could satisfy the integrated optoelectronic modulation (Figure 26a). This synaptic device has a dynamic time response and can provide multi-mode and multi-scale signal processing; meanwhile, due to the ferroelectric and optoelectronic properties of α-In_2_Se_3_, the relaxation time scale and other temporal dynamics of the synapse can be adjusted by optical stimulation (changing light intensity and wavelength) and electrical stimulation (changing pulse amplitude and frequency), which enables the simulation of synaptic properties such as PSC, PPF, PPD, LTP, and LTD (Figure 26b). Based on the above multi-modal tuning, the authors used the synaptic device to build a mixed-signal (multimode) reservoir computing (RC) system with tunable dynamics and multisensory fusion (Figure 26c), which can be used to process multimodal digital data for digital recognition tasks and to make predictions of time series [76].

## 5. Electrolyte Ion-Gated Field Effect Transistors for Building Neuromorphic Systems

In addition to heterojunctions and ferroelectric FETs, electrolyte ion-gated FETs are also commonly used to construct neuromorphic devices; as the name suggests, electrolyte material is used as the gate medium, and the migration behavior of ions in the dielectric material is controlled by the applied gate stimulus to achieve the accumulation or depletion of carriers in the channel. There are three common ion-modulation operation modes: bilayer, electro-chemical doping, and ion encapsulation [112,113]. In terms of transistor modulation, the process of ion generation by this electrolyte under-gate voltage modulation is very similar to the behavior of the biological presynaptic membrane stimulated to produce neurotransmitters; thus, the use of electrolyte ion gate transistors to simulate biological synapses may be more feasible [114]. Although this type of FET has a similar regulatory mechanism to synapses, it is less stable due to the susceptibility of ion production and migration to external influences, and has a slight disadvantage in terms of its nonvolatility and precise conductance regulation comparable to heterojunction structures [115,116]. Though electrolytic ion gate transistors have some of these problems, researchers can attenuate their effects on transistor performance by selecting different channel materials and electrolyte materials because, unlike heterojunctions and ferroelectric field effect transistors (heterojunctions are limited by the need to use thin two-dimensional semiconductor materials, and ferroelectric transistors require materials with ferroelectric polarization effects in the dielectric layer), these types of transistor channel materials and dielectric layer materials are more widely selected, including new two-dimensional materials, various types of organic/inorganic electrolyte dielectrics, etc.; considering different structures and materials can also achieve the preparation of high-performance neuromorphic devices.

**Figure 27:**
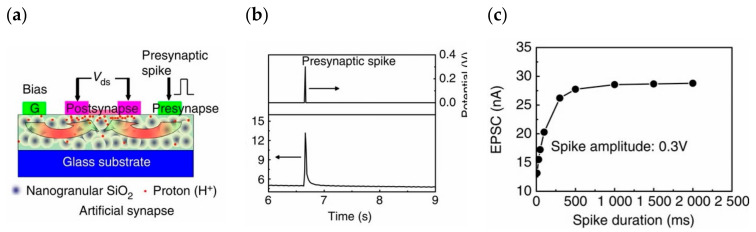
(**a**) Schematic diagram of SiO_2_ double-layer thin film transistor [117]; (**b**) SiO_2_ bilayer thin-film transistor EPSC testing [117]; (**c**) SiO_2_ bilayer thin-film transistor long-time modulation [117]. Copyright © 2014, Nature Publishing Group, a division of Macmillan Publishers Limited. All Rights Reserved.

**Figure 28:**
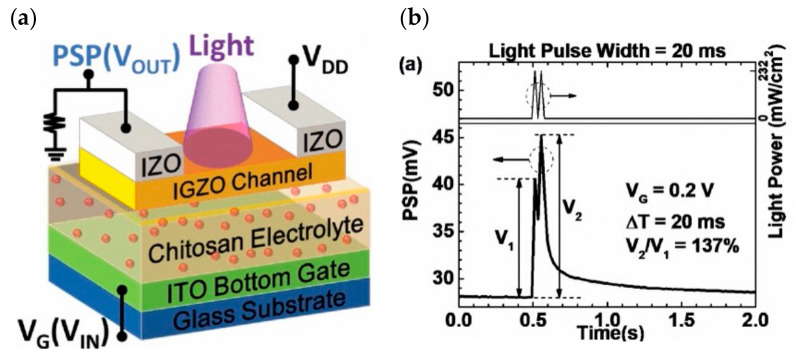
(**a**) Schematic diagram of IGZO double-layer phototransistor [113]; (**b**) IGZO double-layer phototransistor PPF experiment testing result [113]. Copyright © 2018, IEEE.

The first presentation concerns the double-layer mode; in 2014, Qing Wan disclosed oxide-based synaptic transistors that are gated by nanogranular SiO_2_-based proton conductor sheets (Figure 27a). Controlling the electrolyte material to adjust the channel conductance regulates the gate voltage. SiO_2_-based nanoparticle films and chitosan films are used as the gate dielectric, and the voltage applied to the gate is first coupled to the common bottom conductive layer and then to the channel layer. The gate bias is directly laterally coupled into the semiconductor channel via a transverse double-layer capacitor. The device uses IZO as the channel material to realize the preparation of synaptic transistors based on lateral coupling and successfully replicates neuromorphic device characteristics including EPSC, LTP (Figure 27b,c), dynamic filtering, and spatiotemporal correlation signal processing [117]. In 2018, Yi Yang et al. developed an optoelectronic neuromorphic device utilizing IGZO double-layer transistors (Figure 28a). The entire device was constructed on an ITO glass substrate, a solid electrolyte sheet was produced by spin coating on the ITO, and the IGZO channel and IZO electrode were afterward formed by sputtering. The device is able to realize the integrated regulation of photoelectricity, which can effectively simulate important synaptic behaviors such as EPSC, PPF (Figure 28b), and LTP, and can complete the transition of the inhibitory and enhancement effect via gate voltage control, which is significant for the field of photoelectric neuromorphology [113].

**Figure 29:**
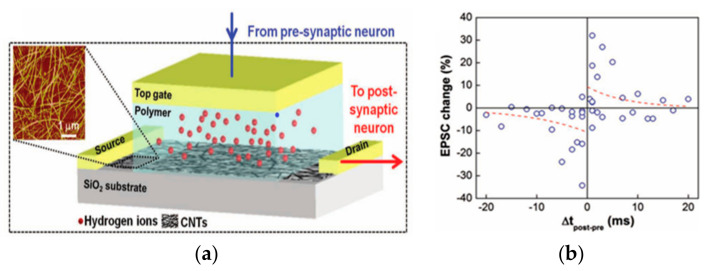
(**a**) Schematic diagram of CNT channel synaptic device [118]; (**b**) STDP characteristics of CNT−channel synaptic devices [118]. Copyright © 2013 WILEY−VCH Verlag GmbH & Co. KGaA, Weinheim.

**Figure 30:**
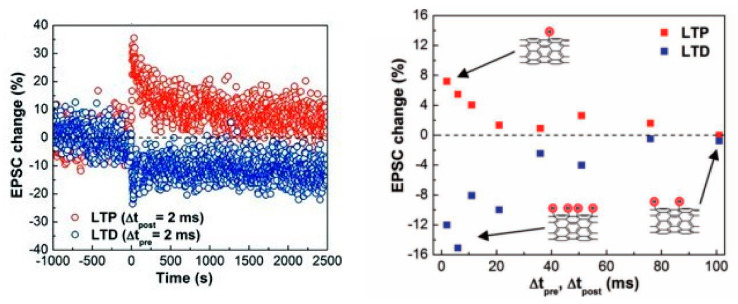
Using EPSC change to reflect the LTP/LTD characteristics in CNT channel synaptic transistors [118]. Copyright © 2013 WILEY−VCH Verlag GmbH & Co. KGaA, Weinheim.

**Figure 31:**
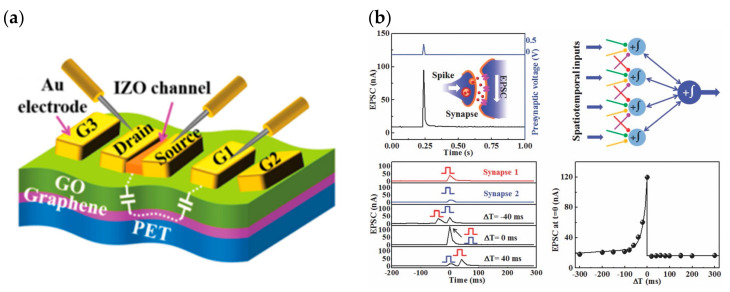
(**a**) Schematic diagram of a flexible neuron device based on graphene/PET substrate material and graphene oxide electrolyte [119]; (**b**) Simulation of EPSC and spatiotemporal correlated logic operations by a flexible neuron device [119]. © 2016 WILEY−VCH Verlag GmbH & Co. KGaA, Weinheim.

**Figure 32:**
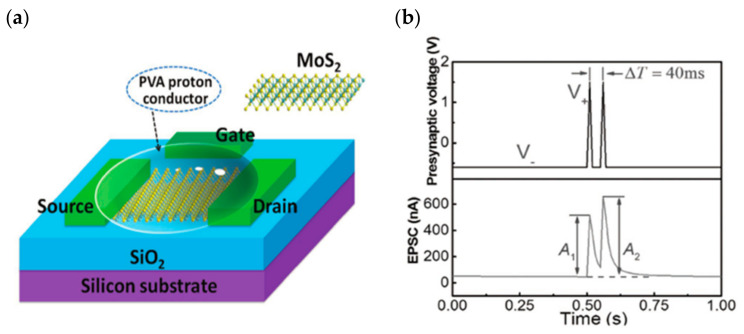
(**a**) Schematic diagram of MoS_2_/Polyvinyl alcohol electrolyte multi-input synaptic device [120]; (**b**) MoS_2_/Polyvinyl alcohol electrolyte synaptic device PPF simulation test [120]. © 2017 WILEY-VCH Verlag GmbH & Co. KGaA, Weinheim.

**Figure 33:**
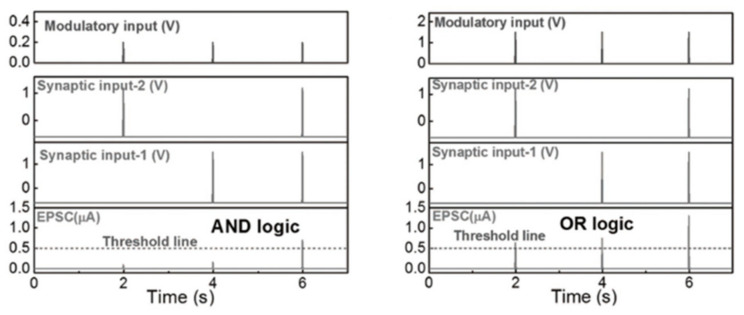
MoS_2_/Polyvinyl alcohol electrolyte synaptic device logic operation simulation [120]. © 2017 WILEY-VCH Verlag GmbH & Co. KgaA, Weinheim.

There have been an equal number of reports on both modes of electrochemical modulation and ionic embedding: first, carbon nanotubes have been shown to have great potential in constructing low-power consumption biological synaptic devices; in 2014, Kim et al. used carbon nanotubes (CNT) as the channel material possessing dynamic logic and learning functions for biological synaptic devices. Additionally, CNT have been shown to have great potential in constructing low-power consumption biological synaptic devices (Figure 29a). In this instance, CNT precipitation was generated by repeatedly dipping the silica substrate into the CNT solution, and a single-walled CNT formed the final channel. This CNT transistor successfully simulated LTP/LTD, STDP (Figure 29b and Figure 30), and is able to conduct some typical biological synaptic learning memory operations, showing its broad future application potential in pattern recognition, intelligent computing, and other disciplines [118]. In 2016, ChangJin Wan et al. constructed a flexible neural device using graphene substrate material and graphene oxide electrolytes (Figure 31a), in which graphene was precipitated on top of the PET flexible substrate using the chemical vapor deposition (CVD) method, and the graphene layer was found to have a small deviation from the mean value and a good uniformity of film resistance. The neuromorphic device with great flexibility and strong electrical characteristics was completed, and the device successfully implemented the logic operation related to spatiotemporal correlation (Figure 31b), which can effectively enhance the development of neuromorphic computing [119]. In 2017, using two-dimensional material MoS_2_ and polyvinyl alcohol electrolyte, Jie Jiang manufactured a neuromorphic synapse with numerous inputs (Figure 32a), in which several inputs can be connected to the MoS_2_ channel, making the transistor’s time-dependent channel conductance modification more realistic. The device successfully replicates synaptic characteristics such as EPSC and PPF (Figure 32b) and can handle pulse modulation-type logic operations and analog multiplication operations (Figure 33) via multiple input gating [120]. In 2020, Da-Shan Shang developed a new device based on polystyrene sulfonate (PEDOT: PSS) film as the channel material and Nafion film as the solid electrolyte for organic electrochemical synapses. The modulation of the channel current of this transistor is affected by the ambient humidity; by controlling the ambient humidity at 26.1%, the device can successfully simulate the synaptic characteristics of STP and PPF, and can realize STP to LTP transition operation. This organic electrochemical synaptic transistor provides a potential impetus for the development of flexible electronic devices and humidity detectors, etc. [121].

**Figure 34:**
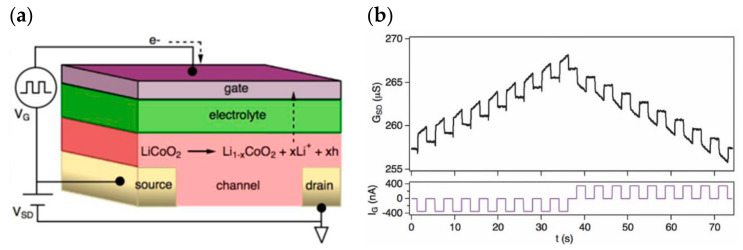
(**a**) Schematic diagram of lithium ion-doped non-volatile electrochemical synapses [122]; (**b**) Simulation of Li^+^ doped non−volatile electrochemical synaptic LTP/LTD properties [122]. © 2016 WILEY−VCH Verlag GmbH & Co. KGaA, Weinheim.

**Figure 35:**
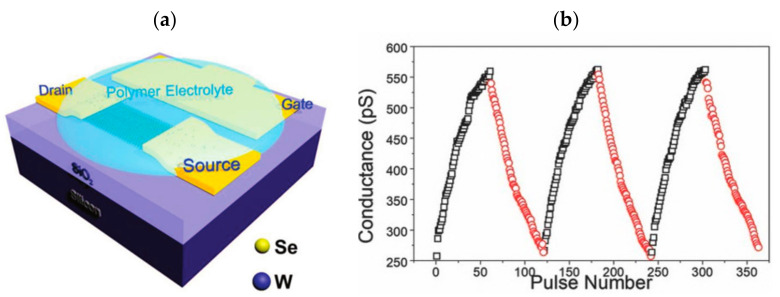
(**a**) Schematic diagram of a synaptic transistor based on a 2D vdw semiconductor [82]; (**b**) Simulation of LTP/LTD characteristics based on 2D vdw semiconductor synaptic transistors. The black represents the long range potentiation and the red represents the long range depression [82]. © 2018 WILEY-VCH Verlag GmbH & Co. KGaA, Weinheim.

Nonvolatile redox reactions are also relevant in electrolyte transistors: in 2017, Alec Talin’s team constructed a solid-state non-volatile electrochemical synaptic device based on lithium ion-doped Li_1-x_CoO_2_ (Figure 34a), which achieves channel resistance regulation through lithium ion insertion/extraction regulation (Figure 34b). The ion migration in this process only needs to cross a low potential barrier, which satisfies the need for low voltage regulation while maintaining non-volatility. The research points to the direction of solid-state non-volatile electrochemical transistors for neuromorphic systems and has the potential to be applied in low-power, high-precision dense array construction [122]. In 2018, Jiadi Zhu et al. developed a related synaptic transistor based on two-dimensional vdw crystal (Wse_2_, NiPS_3_, and FePSe_3_) materials (Figure 35a). Different thicknesses and structures of vdw materials were used, and the synaptic plasticity was then systematically regulated by applying different pulse numbers, durations, rates, and polarities at the gate control side, and finally, the successful simulation of EPSC, PPF, LTP/LTD (Figure 35b), and STDP was achieved. The device also has a very high linearity in the long-range modulation of conductance and an operating power consumption of about 30 fJ per spike pulse, indicating the wide promise of the device for neuromorphic devices [82].

## 6. Memtransistor for Neuromorphic Applications

**Figure 36:**
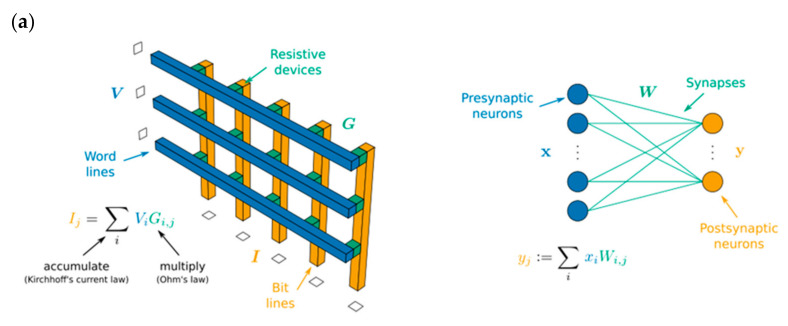

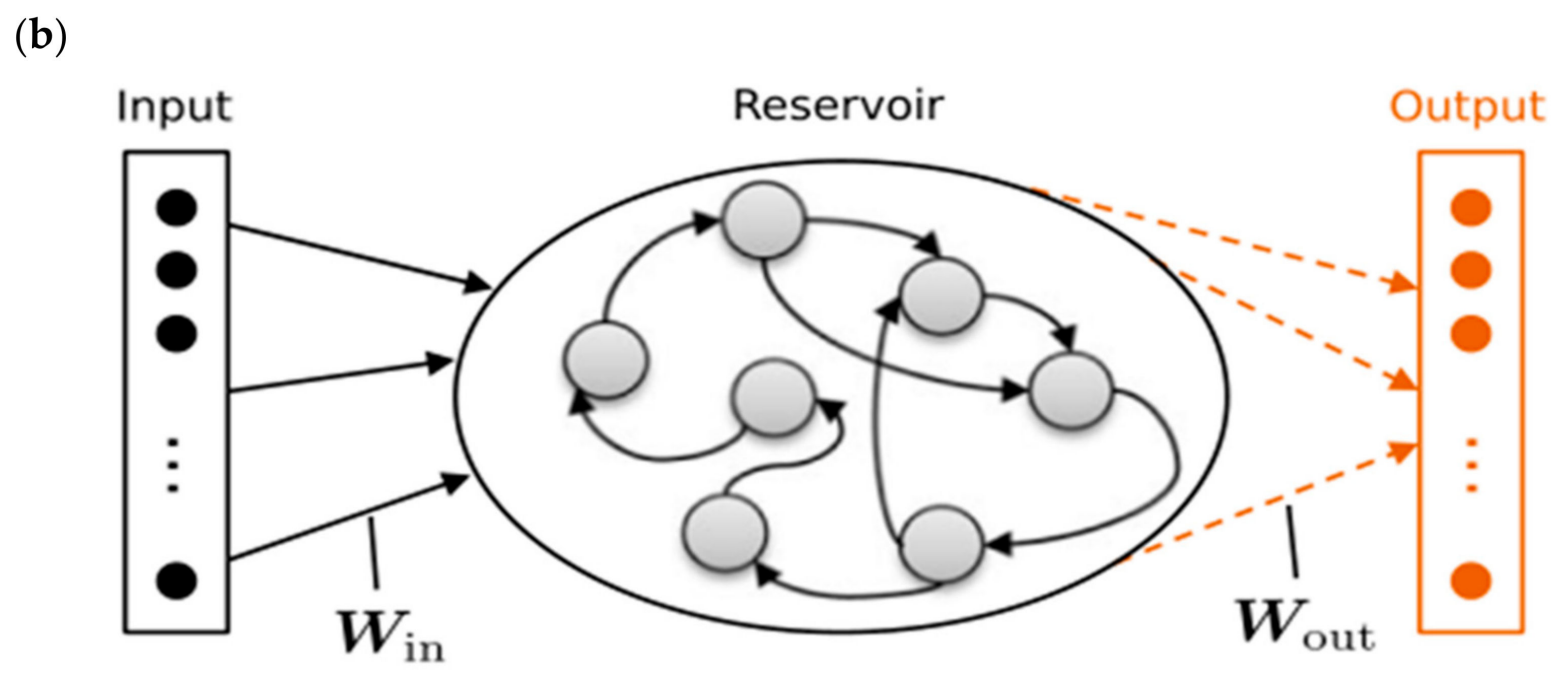
(**a**) Crossbar Arrays structure; the crossbar is a resistive cell, the weight is indicated by the resistance [123]. (**b**) Reservoir computing principal diagram [123].

Emerging artificial intelligence and cloud computing have increasingly invaded people’s daily lives as science and technology have continued to advance. These data-intensive computing approaches are extremely dependent on the current level of computing, in which neuromorphic computing has arisen onto the scene. To address the computing demands of modern science and industry, researchers have developed new computing paradigms based on novel architectures, and neuromorphic computing has been applied in numerous ways. Artificial neural networks, spiking neural networks, convolutional neural networks, and reservoir computing are typical neuromorphic computing techniques. Artificial neural networks are implemented on digital computers, which find it difficult to escape the confines of von Neumann architecture; nevertheless, the hardware-based Crossbar Arrays structure provides a novel way out (Figure 36a); spiking neural networks resemble intra-biological learning, and spike control is used to adjust synaptic weights, which has certain efficiency advantages; convolutional neural networks are frequently used in visual information processing, and the emergence of amnestic devices has started a new chapter in their application in neuromorphic computing. Reservoir computing is an extension of recurrent neural networks with short-term memory and nonlinear modulation capabilities. Reservoir computing is comparable to memristors and has paved the path for its use in neuromorphic computing (Figure 36b) [85,123,124]. Today, the design of synaptic hardware devices has reached a certain level of development, and scientists are actively considering how to transfer the synaptic properties of hardware to neuromorphic computing to meet the needs of dynamic visual sensing, image recognition, and information encryption.

**Figure 37:**
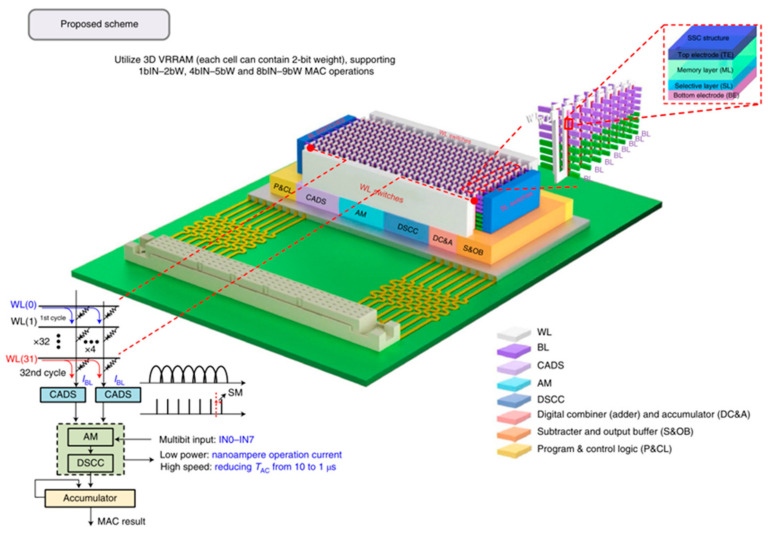
High-precision NVCIM solution based on MLSS 3D VRRAM [126]. Copyright © 2022, Qiang Huo et al.

**Figure 38:**
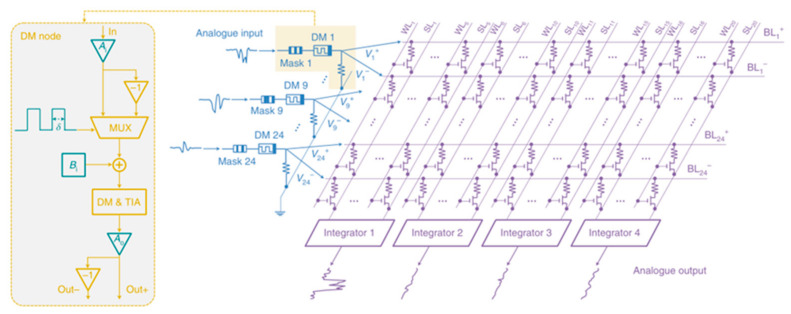
Architecture of the RC hardware system [127]. Copyright © 2022, Yanan Zhong et al., under exclusive license to Springer Nature Limited.

**Figure 39:**
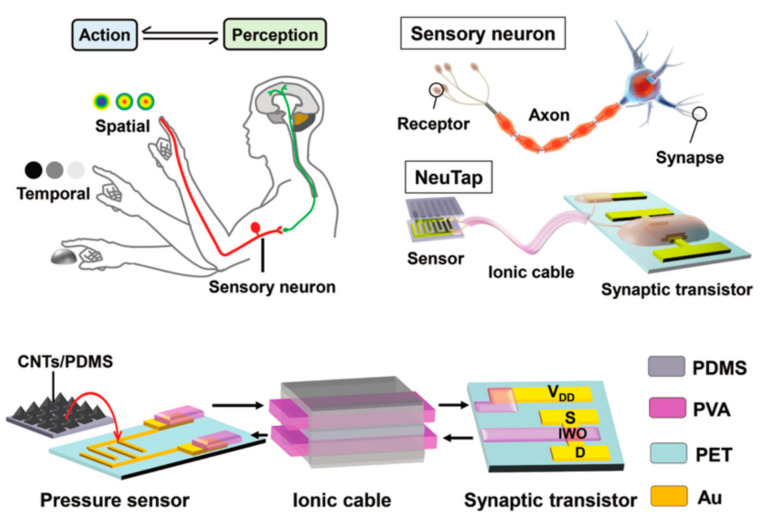
Schematic of the conceptual design of NeuTap [129]. © 2018 WILEY-VCH Verlag GmbH & Co. KGaA, Weinheim.

**Figure 40:**
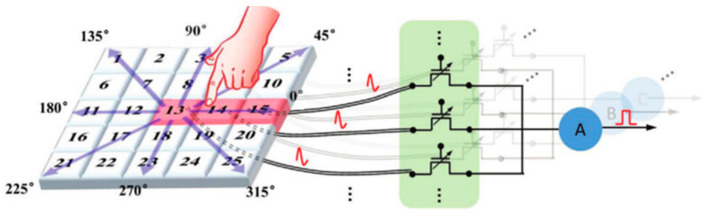
Artificial tactile sensor arrays, where tactile sensors receive spatiotemporal tactile signals and input them into synaptic arrays, which are then processed and recognized by the SNN, and finally output spikes by the output neurons [130]. © 2020 Wiley-VCH GmbH.

The concept of the memristor has been explored since its inception, and researchers have explored its great potential for neuromorphic networks based on its unique resistive memory properties [125]. The two-terminal devices are usually capacitive, with the upper and lower electrodes holding the resistive material, making them easier to design and fabricate. This type of device has received a lot of attention in the early days, and researchers have invested a lot of effort and achieved remarkable results. For example, in 2022, Feng Zhang’s group built a complete in-store computing circuit based on a two-terminal random memory device, and the circuit structure achieved a high energy efficiency ratio of up to 62.11 TOPS/W and a bit density of 58.2 bit/um^2^ (Figure 37) [126]. Based on reservoir computing technology, the computational system built with two-terminal amnesia was able to achieve efficient signal processing with an accuracy of 96.6% and 97.9% in temporal arrhythmia detection and spatiotemporal dynamic gesture recognition tasks, conducted by the Huaqiang Wu group at Tsinghua University (Figure 38) [127]. While two-terminal amnestic devices are having an impact in the hands of researchers, three-terminal (multi-terminal) transistor amnestic devices are seen as another important branch for future neuromorphic device development due to their unique gating mechanism and mature theoretical foundation. As mentioned above, the process of controlling the various states of a transistor device through certain conditions of the gate is very similar to the process of neuromorphic devices that release neurotransmitters and regulate the strength of connections [128]. Transistors are also able to achieve larger current switching ratios through gate control, maintaining large on-state currents to meet faster operation while maintaining very low current levels in the off-state, thus reducing power consumption, which is an advantage in building large-scale, highly integrated neural networks in the future. In 2018, Changjin Wan built a neuronal device based on ion channels for NeuTap (a neuromorphic tactile processing system that can receive external information for perceptual learning), resistive pressure sensors, and synaptic transistors that enable the basic simulation of bio-sensory neuronal function, a basic simulation of neuronal function (Figure 39). The resistive pressure sensor turns the pressure stimulus into an electrical signal, and the ion conductor conveys the electrical signal to the synaptic transistor via interface ion/electron coupling to complete the transmission and transformation of the signal. Several touch modes are available on the neural device, which is also capable of distinguishing between distinct spatiotemporal signal properties and external inputs. After multiple training sessions, the accuracy of the device’s recognition is enhanced, and the device’s characteristics demonstrate a strong resemblance to perceptual neurons, making it suitable for future use in fields such as neuromorphic artificial skin and brain-computer interfaces [129]. Progress has also been made in pattern recognition and learning associations. In 2013, Yukihiro et al. from Japan built the first neuromorphic network for pattern recognition using a three-terminal non-volatile memory device. The neuromorphic chip was based on CMOS circuit technology and used ferroelectric-like memristor devices, which were trained to recognize incomplete pattern inputs through correlation learning matrices [42]. In 2020, Yue L et al. created a 32 × 32 array of electrolyte gate transistors with a number of advantageous properties, including quasi-linearity, good durability, high switching speed, low readout conductance, and low power consumption, enabling the array to achieve efficient learning and recognition. Based on this, the authors built a hardware version of a spiking neural network (SNN) for spatiotemporal information processing (Figure 40), which may be applied to motion direction recognition in tactile sensing systems, creating a new application scenario for future neuromorphic computing [130]. In 2022, the team led by Tian revealed significant progress in their modeling of the biological brain’s associative skills. The team constructed a perceptual learning network based on a three-terminal device to mimic the weight regulation process of integrate and fire (IF) neurons during accumulation and release in living organisms; the connection strength was varied by modulating the conductance via the gate voltage, and IF neurons were then used to recall the training object. Using partial digital information, the circuit network successfully achieves an associative recall of all digital images from 0 to 9 following suitable training [131].

**Figure 41:**
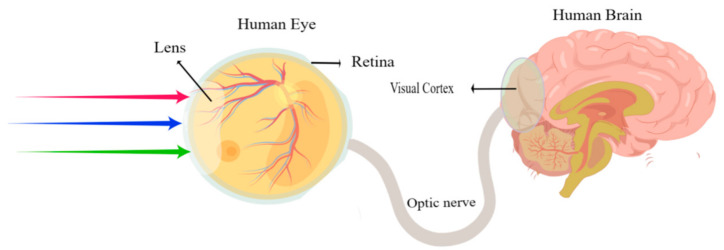
Schematic diagram of the human vision system architecture.

**Figure 42:**
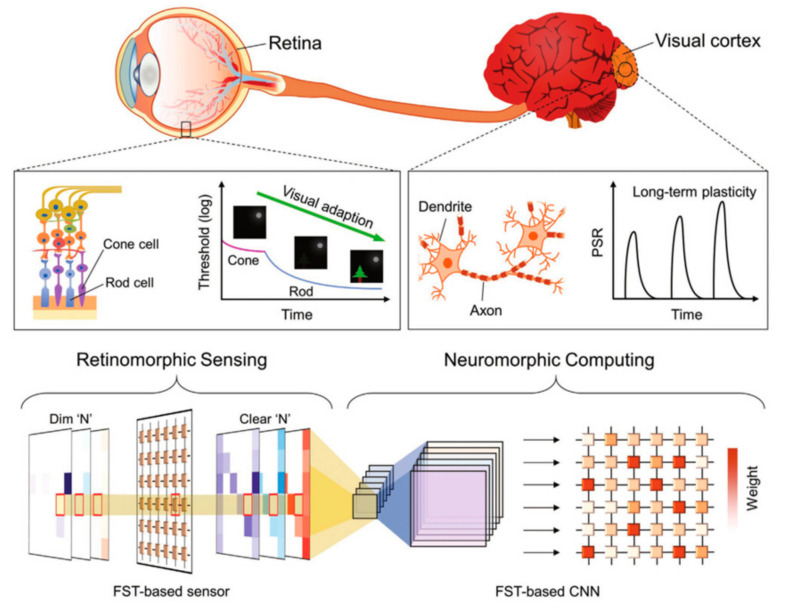
Hardware implementation of FST-based NMVS [134]. © 2022 Wiley-VCH GmbH.

**Figure 43:**
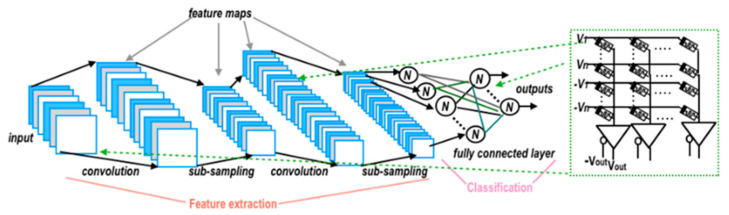
CNN computation flow diagram [135]. Copyright © 2020, IEEE.

Neuromorphic networks inspired by living organisms have received a great deal of attention for their higher efficiency [117] and lower power consumption than traditional vision systems in machine vision, and has led to many advances in neuromorphic vision [1,35]. The human visual system consists of the eyes, the optic nerve network, and the cerebral cortex, as shown in Figure 41. Visual information from the outside world is first received by the eye, where the signal is focused and adjusted by the lens, and then transmitted to the retina, which perceives and pre-processes the visual signal, extracting relevant information from it. The processed information is then passed through the optic nerve network and finally conveyed to the visual cortex of the brain for final processing to form vision [132,133]. Throughout the process of vision formation, the retina extracts the key features of the signals as they are received at the front end, thus eliminating the need for a multitude of redundant data from the perceptual part of the visual information and greatly reducing the pressure of data transmission, also with a much faster rate than any other visual sensing system available today. In 2022, Yuchen Cai et al. constructed a neuromorphic machine vision system (NMVS) (Figure 42) that integrates a front-end retinal morphological sensor and a back-end convolutional neural network (CNN) based on a single ferroelectric semiconductor transistor (FST) device architecture, allowing it to display broadband retina-like light adaptation, a large dynamic range, a programmable operation, and an accuracy of up to 93.0%, indicating its great potential for artificial biological vision [134]. A hardware solution for the CNN mentioned in that report is shown in Figure 43, containing four parts: a convolutional layer, a pooling layer, an activation function layer, and a fully connected layer. The architecture is mainly divided into a feature extraction part with convolutional and down-sampling layers, and a classification part [133]. The convolutional layer is obtained by a sliding window-by-window calculation of the convolutional kernel on the upper input layer. Each parameter in the convolutional kernel is equivalent to a weight parameter in a traditional neural network and is connected to the corresponding local pixel; the result on the convolutional layer is obtained by multiplying the sum of each parameter of the convolutional kernel with the corresponding local pixel value [135]. In convolutional neural networks, lower-level convolutional layers extract low-level features, such as edges, lines, and corners, and higher-level convolutional layers extract higher-level features; the data processing and circulation patterns of the two are very similar. In 2020, Feng’s team used the WSe_2_/h-BN/Al_2_O_3_ heterostructure to simulate bipolar cells and photoreceptors, integrating both types of cells through the heterostructure to make the vision system more compact; the pixel array constructed by the device built a reconfigurable vision sensor with an accuracy of 100% in less than 10 training cycles [63]. In 2021, Zhou’s team used a device constructed from the WSe_2_/h-BN heterostructure to simulate the positive/negative photocurrent response of bipolar cells in the retina, and then used the device to build an integrated retinal simulation device to achieve 100% separation detection of moving trichromatic trolleys without ghosting [62]. In 2022, Chai et al. used a double layer of MoS_2_ phototransistors to simulate horizontal and photoreceptor cells in the retina; the different states of the transistors were modulated by a trap capture mechanism, allowing the phototransistor array to display both light/dark adaptation states, creating an effective sensing range (up to 199 dB) and enabling image contrast enhancement [96]. In addition, visual synaptic devices have also been reported, such as the aforementioned Changhwan Choi et al., who constructed an optic nerve synaptic device using h-BN as a channel modulation layer to regulate the conductance of the WSe_2_ channel, demonstrating a visual sensing device with synaptic and optical sensing capabilities [8]. In summary, there are two main types of neuromorphic vision sensors based on some novel devices: (1) dismembering the parts of the human visual system to analyze their working mechanisms and constructing corresponding architectural devices with a view to reproducing their functions; (2) building artificial optoelectronic synaptic devices to simulate the workings of visual neural networks. In comparison, the first option is closer to the one in which researchers simulate visual sensing through human organs and cells, and it is easier to imitate the working mechanism of an individual particular part; the disadvantage lies in the fact that scientists today do not have a thorough understanding of the mode of operation of various types of working cells, but only a general description of their role and macroscopic regulatory mechanisms at the systemic level. As far as optoelectronic synaptic devices are concerned, FET-based optical signal modulation synaptic devices can be used as large bandwidth, low interconnection energy devices and help build new neural network architectures. With the current trend in brain-like and neuromorphic computing, visual sensors built with synaptic and neural components may be more easily integrated and coupled into various neuro-mimetic circuits in the future [40,41]. However, there are still some challenges that need to be overcome, such as device reliability, reproducibility, etc. Only a small fraction of excitatory synaptic functions have been simulated, and more researchers are expected to study them in the future to bring us more results for the benefit of humanity.

## 7. Conclusions and Future Perspectives

In this review, the new emerging materials used as the channel materials and gate stack layers used as modulation mediums for memtransistor fabrications are discussed. First, we discuss the resistive switching mechanisms related to the different device fabrications. The key characteristics of memtransistors are demonstrated in Section 2. Then, we focus on the main emerging trends in memtransistors, such as 2D materials stack-based memtransistors, the charging carrier in the interface, and the stacking sequence, which have an important influence on the device characteristics. As for the voltage-tunable ferroelectric domain structure memtransistors, the resistive switching characteristics result in the ferroelectric gate modulation. The role of ferroelectric polarization on the channel materials is discussed in detail. How to design the structure of ferroelectric synapse devices, and some technological measures to improve the properties of ferroelectric media (for example, annealing after the precipitation of ferroelectric medium) also have potential influence on the resistive switching characteristics. It is worth pointing out that the hafnium-based material’s compatibility and excellent ferroelectricity enable the transistor to be continuously modulated, giving the device greater potential in future neuromorphic applications. At the same time, the non-negligible depolarization effect due to the non-ideal electrodes of ferroelectrics and the polarization instability should be given more attention. Transistors in the form of electrolyte-ion grids rely on various types of ion migration to operate in a manner that is very similar to that of biological synapses, and therefore, have a natural advantage in simulating biological synaptic operations and behaviors. In this context, important artificial synapse characteristics, such as paired-pulse facilitation (PPF), spike-rate-dependent characteristics (SRDP), and spike-timing-dependent plasticity (STDP), potentiation, and depression behavior, are demonstrated.

New artificial synaptic devices indicate a new route for neural morphology computing. However, there are still a number of issues to be resolved in the process of preparing various synaptic devices. Due to their exceptional external sensitivity, two-dimensional materials have been widely utilized in the development of memristor-based synaptic devices. However, the industrial preparation technology for large-scale, high-quality, and wafer-level two-dimensional materials is still very complicated; the bonding, interface optimization, and dependability of two-dimensional materials with other types of semiconductor materials require additional research. These issues impede the large-scale integration of two-dimensional synaptic device series arrays. The discovery of ferroelectric polarization in hafnium materials enables the incorporation of ferroelectric transistors into the existing mature CMOS technology. However, at smaller dimensions, the ferroelectric characteristics of hafnium-based materials are significantly impacted by oxygen vacancies, interface traps, etc., which have a significant impact on the normal operation of devices. The use of electrolyte materials as gate media can improve the capacity to control carriers in the channel, but in the presence of a rapidly varying electric field, the protons in the gate media are unable to respond in time, hence slowing the device’s response speed. In addition to the inherent drawbacks of various types of synaptic devices, synaptic devices face some common obstacles: the simulation degree of existing artificial synaptic devices for the synaptic mechanism is still relatively shallow, the majority of devices can only simulate basic synaptic behavior, and the cognitive level of the higher-level plasticity-containing learning mechanism is low. Existing reported synaptic devices have no benefit in terms of device performance over traditional devices with established technology. Researchers must investigate the stability of gadget function and the ongoing shrinking of feature size. In addition, there are no industry-recognized evaluation standards for comparing the performance of various types of synaptic devices. In the design of circuit networks, the emergence of multi-terminal memtransistors gives a novel solution to the problem of series current leakage channels in the two-terminal device circuit networks. However, in order for the synaptic device to accurately and efficiently operate, it must be subject to stringent and stable control, so it is necessary to implement a larger-scale peripheral circuit, which reduces the working efficiency and scalability of the circuit and imposes stricter requirements on how to ensure the uniformity of device performance during device preparation [30,61,85,86,136].

Overall, emerging memtransistors present exciting opportunities to improve device performance and new operation mechanisms for neuromorphic system applications. Research at the material, device, and system levels should be simultaneously adopted. The future of neuromorphic computing is expected to be used in all kinds of life and work scenarios, and high efficiency and low power consumption are still the goals of future circuit system development, applied to future human intelligent living patterns and to improve our living conditions.

## Figures and Tables

**Table 1:** Some parameters related to artificial synaptic devices that have been reported.

Device Type	Structure	Channel Material	Voltage	Number of States	Ion/Ioff	Hysteresis	Linearity	Power Consumption	Endurance	Dynamic Range	Reference
vdw heterostructure FET	h-BN/WSe_2_/Al_2_O_3_/BP	WSe_2_/BP	14 V	26			0.99577		>1.5 s		[62]
	WSe_2_/BN/Al_2_O_3_	WSe_2_	9 V				*		>1 s		[63]
	WSe_2_/WCL/h-BN	WSe_2_	0.3 V	599			1.4	66 pJ			[64]
	MoTe_2_/PdSe_2_/Si, SiO_2_	MoTe_2_	10 V				1				[65]
	WSe_2_/h-BN		20 V	130	1.1 × 10^6^			>1 nJ	4.5 × 10^4^ s		[66]
	MoS_2_/PTCDA	MoS_2_	12 V	50			*	10 pJ		14.0 dB	[67]
	SnSe/BP/POx		20 V	20				~nJ			[68]
Ferroelectric transistors	Si, SiO_2_/TiO_2_/PZT/MoS_2_	MoS_2_	2.9 V		10^4^	>4 V	*		10^4^		[69]
	Poly-GeSn/Ta_2_O_5_/HfZrOx/TiN	GeSn	8 V	80						9.6 dB	[70]
	HZO/Si	Si	3.2 V	32	10^6^	2.5 V	1.73/1.86		10^4^		[71]
	HfO_2_/SiON/Si	SiON	3 V	>6		0.75 V					[72]
	SrRuO_3_/PZT/ZnO	ZnO	6 V				~1		>10 years		[73]
	Al/IGZO/HZO/TiN	IGZO	6 V	64	10^4^	3 V	0.8028/0.6979		10^3^	10 dB	[74]
	Al2O_3_/WOx/HZO/TiN	WOx	3 V	16		4 V	*		1500 s		[75]
	α-In_2_Se_3_/HfO_2_, Si	In_2_Se_3_	2 V		10^6^	3 V					[76]
	PVDF, PVP/P(IID-BT)	P(IID-BT)	30 V		10^5^	20 V	*	75 pJ	10^4^	81 dB	[77]
Memtransistor (Ion Transportation, Electrochemical, etc.)	MoS_2_/SiO_2_	MoS_2_	20 V	256			0.99	17 pJ	10^4^	34.9 dB	[78]
	CNT/SiOx/Au/SiOx/Pd	CNT	8 V	120						50 dB	[79]
	LiClO_4_/α-MoO_3_/Si, SiO_2_	α-MoO_3_	2.5 V	50		1.5 V	*	0.16 pJ		1.62 dB	[80]
	Pt/Si/Li_3_POxSex/LiCoO_3_		1.5 V	90					7 × 10^2^	19 dB	[81]
	WSe_2_, NiPS_3_, and FePSe_3_/SiO_2_	WSe_2_, NiPS_3_, and FePSe_3_	1.2 V	60			*	30 fJ		2.3 dB	[82]
	WSe_2_/WO_3-X_	WSe_2_	4 V	30			*			100 dB	[83]

“*” means a good linearity but no specific value given.

## Data Availability

All the date is from the references we have cited.
